# Small mammal owners’ experiences of housing challenges and animal welfare: A COM-B and word frequency analysis

**DOI:** 10.1017/awf.2025.10034

**Published:** 2025-08-21

**Authors:** Grace Carroll, Kerry Taylor, Claire Stallard, Alison Wills

**Affiliations:** 1 School of Psychology, Queen’s University Belfast, UK; 2 Blue Cross, Oxfordshire, UK; 3 Hartpury University and Hartpury College, UK

**Keywords:** Animal welfare, COM-B, companion animal, housing, pet, rabbit, rodent

## Abstract

Small mammals are particularly dependent on owner-provided housing and husbandry yet are frequently kept in conditions that do not meet their welfare needs. This study used the COM-B model (Capability, Opportunity, Motivation = Behaviour) to identify behavioural drivers influencing housing provision among 723 UK small mammal pet owners. This model of human behaviour proposes that behaviour occurs when individuals have the capability, opportunity, and motivation to act. Owners of the eight most commonly kept small mammal species were surveyed: rabbits (*Oryctolagus cuniculus*), guinea pigs (*Cavia porcellus*), hamsters (*Cricetinae*), gerbils (*Gerbillinae*), rats (*Rattus norvegicus*), mice (*Mus musculus*), chinchillas (*Chinchilla lanigera*), and degus (*Octodon degus*). Opportunity, particularly the availability of suitable enclosures, emerged as the primary barrier, while Capability and Motivation were identified as facilitators, with most pet owners willing and able to provide good levels of welfare. Owner approaches to assessing health and welfare at home were examined through qualitative word frequency analysis, with responses mapped to the Five Domains model. This analysis focused on rabbits, guinea pigs, rats, and hamsters due to limited data availability for other species. Overall, behavioural indicators were most commonly used to identify positive health and welfare, while nutritional and physical signs were cited most frequently for negative states. Changes in eating behaviour were the most frequently cited indicators of ill health or poor welfare across all four species, suggesting this may serve as a practical health and welfare indicator for owners. Improving access to suitable housing and further exploring eating behaviour as an early health and welfare indicator may together support better husbandry for small mammal pets.

## Introduction

Small mammal species are becoming increasingly popular as companion animals (McLaughlin & Strunk 2016; Díaz-Berciano & Gallego-Agundez 2024). A UK-wide pet census by Blue Cross (2024) found that 9% of respondents owned rabbits (*Oryctolagus cuniculus*), and 9% owned other small pets, with guinea pigs (*Cavia porcellus*) and hamsters being most popular, followed by rats (*Rattus norvegicus*), gerbils, chinchillas, degus and mice (*Mus musculus*), respectively. This represents hundreds of thousands of animals, including approximately 1 million rabbits, 700,000 guinea pigs, and 600,000 hamsters (UK Pet Food 2024). Despite their popularity, there is surprisingly limited information available regarding the natural behaviour, health, welfare, care and housing requirements of these species (Harrup & Rooney 2020; Mee *et al.* 2022; Hedley *et al.* 2023; Gilhofer *et al.* 2024; Schneidewind *et al.* 2024). Small mammal pets belong to different orders; the order, *Rodentia* includes rodents like mice, rats, hamsters and gerbils, while rabbits belong to the order, *Lagomorpha (*Allaby 2003). Together, these orders make up the mammalian clade, *Glires* (Yeates & Baumans 2019). Within this clade, there are various wild and domesticated species, subspecies, and breeds, each with distinct needs (Lonstein & De Vries 2000; O’Neill *et al.* 2022). Some species were domesticated for use as a food source or for their fur, while others were bred for scientific research purposes, or purely for aesthetics (Mitchell 2009; Yeates & Baumans 2019), further contributing to their diversity (Linderholm & Larson 2013; Saré *et al.* 2021). Despite these differences, small mammal pets are often treated as a homogeneous group. For example, many small mammal enclosures are labelled for ‘rodents and small animals’ rather than a specific species (Bläske *et al.* 2022).

### Small mammal housing

There is evidence to suggest that pet rabbits and small rodents are often housed in inappropriate enclosures. For example, Rooney *et al.* (2014) surveyed 1,254 rabbit owners across South-West, North-West and Eastern England and found that 27.5% of rabbits were housed in enclosures that limit natural behaviour. Furthermore, 43.5% of rabbits, a social species, were housed singly, and a small number were housed with predator species (e.g. domestic cats [*Felis catus*]). Similarly, Mee *et al.* (2022) found that 31.2% of rabbits lived in inadequate housing, with half being housed alone. Harrup and Rooney (2020) also identified poor housing practices in guinea pig owners, with 21.4% of guinea pigs being housed alone, and 18.2% being housed in enclosures smaller than those recommended by the British Cavy Council (Neesam 2015). In addition, commercially available enclosures may not always meet optimal housing standards. For example, Bläske *et al.* (2022) assessed the suitability of small pet products in Germany, including enclosures, bedding, and accessories. Criteria were created based on animal welfare legislation and animal welfare organisation guidelines. Between 50 to 100% of species-specific enclosures were evaluated as being unsuitable for the specific animal in question as regards their welfare (Bläske *et al.* 2022). These ongoing issues may stem, in part, from a lack of research into species-specific housing needs, making it difficult for both manufacturers and owners to make informed decisions. Much of the research on small mammal housing has been conducted for laboratory animals, where animal welfare competes with other priorities, including financial and scientific considerations (Mazhary & Hawkins 2019; Harrup & Rooney 2020; Neville *et al.* 2022). However, the limited evidence that does exist suggests that enclosures should be as big as is feasible; those that are too small may restrict the ability of the animal to perform natural behaviours and can increase inactivity (Dixon *et al.* 2010; Hedley *et al.* 2023). Furthermore, we know that many *Glires* are social animals and should be housed in pairs or small groups (Bläske *et al.* 2022; Gilhofer *et al.* 2024; Schneidewind *et al.* 2024), while recent evidence suggests that, although some species, such as Syrian hamsters (*Mesocricetus auratus*), have been traditionally housed alone, all hamster species may require solitary housing (Ross *et al.* 2017; Hedley *et al.* 2023).

Appropriate housing is of concern to small mammal owners; in their Big Pet Census, Blue Cross (2024) found that 8% of respondents identified “ensuring pets have adequate housing” as their top welfare concern. However, this may relate to their own ability to provide housing, the availability of housing on the market, or something else. According to Section 9 of the UK Government Animal Welfare Act (2006), pet owners are responsible for meeting their animals’ day-to-day needs. This is especially important for animals kept in enclosures, where they rely entirely upon humans for food, water, and care. Furthermore, inappropriate husbandry in small mammals is often linked to poor health, meaning a lack of suitable housing has a significant impact on animal welfare (Wills 2020). Considering this, it is important to understand the barriers that prevent owners from providing appropriate housing, and to determine whether these relate to intrinsic factors (e.g. knowledge, habits), external constraints (e.g. availability of suitable enclosures, social norms), or a mixture of both.

### Understanding owner behaviour: Intrinsic and extrinsic barriers

In recent years, animal welfare science has moved away from focusing solely on the attitudes and intentions of those responsible for the care of animals, to adopting broader human behaviour change frameworks that consider a wide range of influencing factors (Carroll & Groarke 2019; Cornish *et al.* 2019). For example, while pet owners may intend to provide suitable housing, habits, the social environment or the availability of resources may also determine their behaviour, factors which, although touched upon in earlier attitude models (via normative and control beliefs) are treated more explicitly and independently in newer frameworks. In order to change human behaviour to improve animal welfare, the barriers and facilitators associated with a desired outcome must be identified (Michie *et al.* 2011; Carroll *et al.* 2021). This can be done using the Behaviour Change Wheel (Michie *et al.* 2014); a framework that was originally used by Health Psychologists to change human behaviours, such as smoking and physical inactivity (e.g. Fulton *et al.* 2016; Truelove *et al.* 2020). Barriers and facilitators can be identified using the COM-B model (Capability-Opportunity-Motivation = Behaviour) which can, in turn, be mapped to viable solutions known to be successful in changing human behaviour (Michie *et al.* 2014). This model of human behaviour proposes that behaviour occurs when individuals have the Capability, Opportunity, and Motivation to act. Each of these components can be divided into two subtypes, resulting in six categories in total (Michie *et al.* 2014). Capability includes both physical capability (e.g. strength, dexterity) and psychological capability (e.g. knowledge, cognitive skills). Opportunity is divided into physical opportunity (e.g. time, resources) and social opportunity (e.g. cultural norms, social expectations). Motivation is comprised of reflective motivation (e.g. conscious planning, beliefs, intentions) and automatic motivation (e.g. habits, emotional responses, impulses). To the authors’ knowledge, this approach is yet to be used in the context of rabbit and small rodent companion animal housing.

### Welfare assessment of small mammals

Similar to housing requirements, methods of assessing rabbit and small mammal welfare are under-developed. Cohen and Ho (2023) conducted a systematic review of rat, mouse, guinea pig and rabbit welfare indicators and found there to be a lack of focus on direct welfare assessment methods. Similar to housing guidelines, most welfare measures are found within the grey literature (e.g. veterinary textbooks, animal welfare organisation materials), with few coming from the scientific literature. Welfare assessment measures that involve direct assessment of the animal more accurately reflect the welfare state than indirect resource-based measures alone (European Food Safety Authority [EFSA] 2012). For example, assessing enclosure size is a resource-based measure, while assessing coat quality or animal behaviour would be animal-based measures. While physiological measures are less practical to assess and often require a level of expertise and skill (Cohen & Ho 2023), physical and behavioural measures are more accessible to pet owners. Recently, James and Wills (2025) surveyed 1,700 guinea pig, hamster, rat, gerbil and mouse owners and found an association between owners’ perceived confidence in identifying illness, and their actual accuracy when asked to recognise clinical signs and behaviours indicative of ill health. Although this association was weak, it suggests that small mammal pet owners may have some capacity to accurately assess their animals’ welfare status. This is particularly important given that small mammals are less likely to receive veterinary care compared to species such as cats and dogs (*Canis familiaris*) (Fox & Neville 2024). Furthermore, veterinarians often have limited training as regards the treatment of exotic species and have reduced confidence in their ability to provide adequate care (Grant *et al.* 2017; Wills & Holt 2020; Espinosa García-San Román *et al.* 2023). The brief time spent with each animal may also hinder thorough assessments (Robinson *et al.* 2014). In order to improve small mammal welfare, it is important to understand how owners assess their pets’ health and welfare in the home.

The aims of the current study were thus to: (a) assess barriers to, and facilitators of, provision of suitable housing for pet rabbits and rodents in the UK; and (b) determine key positive and negative health and welfare indicators in pet rabbits and rodents, according to their owners.

These aims allow for an assessment of both welfare inputs, such as the physical environment provided, and welfare outputs, such as the owners’ ability to recognise indicators of welfare status.

## Materials and methods

### Ethical considerations

Hartpury University Ethics Committee (ETHICS2021-113) approved this study on 27 July 2022.

### Study design and recruitment

A cross-sectional descriptive study design was used, with quantitative and qualitative elements. An online survey was disseminated by Blue Cross through paid Facebook advertising. UK-based owners of the eight most commonly kept species were targeted: rabbits, guinea pigs, hamsters, gerbils, rats, mice, chinchillas and degus. Links to the survey were also shared via relevant Facebook groups. The survey was hosted on Qualtrics and was live between the 23 November 2022 and 6 January 2023.

### The survey

A detailed survey was distributed to collect information regarding owner and pet demographics, housing, enrichment, diet, bedding, animal welfare, and barriers to appropriate housing. The survey was designed to enable reporting the key barriers to provision of suitable housing, and participants’ qualitative assessments of their pets’ welfare and to be completed for one animal only. Participants were instructed to select one species, and if they owned multiple animals of the same species, they were to choose the individual whose name appeared first in the alphabet. Participants were directed automatically to the branch of questions relevant to their selected species and could choose to leave certain questions blank should they so desire.

### Barriers to provision of suitable housing

Participants were required to rate 51 statements on a 5-point Likert scale (1 = strongly agree, 2 = agree, 3 = neither agree nor disagree, 4 = disagree, 5 = strongly disagree). These statements were based on a review of the literature and the expert knowledge of the authors and were framed by the COM-B and Theoretical Domains Frameworks (Cane *et al.* 2012; Michie *et al*. 2014). The COM-B model is used to gain an understanding of behaviour in-context. For a given behaviour to occur, there must be the ‘Capability’ to do it, the ‘Opportunity’ for it to occur, and the ‘Motivation’ to perform the behaviour (Michie *et al.* 2014). The Theoretical Domains Framework (TDF) is made up of 14 domains that help explain what influences behaviour; ‘Knowledge’, ‘Skills’, ‘Memory, attention and decision processes’, ‘Behavioural regulation’, ‘Social/professional role and identity’, ‘Beliefs about capabilities’, ‘Optimism’, ‘Beliefs about consequences’, ‘Intentions’, ‘Goals’, ‘Reinforcement’, ‘Emotion’, ‘Environmental context and resources’, and ‘Social behaviour’. The TDF sits under the COM-B model (Cowdell & Dyson 2019). For example, ‘Knowledge’ and ‘Skills’ sit under ‘Capability’ and ‘Belief about consequences’ sits under ‘Motivation’. Questions were posed under each of the 14 domains. While each item was mapped to a single COM-B domain for clarity, we acknowledge that some constructs, particularly belief-based statements, may align with more than one domain. For example, normative beliefs shaped by social influence were classified under ‘Social opportunity’, though they also reflect aspects of ‘Motivation’ (Whittal *et al.* 2021).

Descriptive statistical analyses were carried out in SPSS, version 29.

### Qualitative health and welfare assessments

Participants were asked to write down up to three signs that they thought indicated their pet’s positive or negative health and welfare status (“I know when my [species] is happy/healthy when…” and “I know when my [species] is unhappy/ill when…”).

### Word frequency analysis

Word frequency analysis was utilised to determine the most common words used to describe signs of positive and negative health and welfare. Word frequency analysis allows patterns to be easily identified and can decrease bias in interpretation of the data (Onwuegbuzie & Leech 2007; Feng & Behar-Horenstein 2019). Words were required to contain three or more letters, and were initially grouped with stemmed words, for example, ‘hide’, ‘hiding’ and ‘hides’). Stop-words like ‘a’, ‘for’, and ‘have’ were excluded (Baradad & Mugabushaka 2015) as they do not contribute meaning to the descriptions given by pet owners. The 25 most frequently used words were then assessed manually, with synonyms being grouped together (e.g. ‘lethargic’, ‘tired’). From this, the ten most frequently used words, and associated synonyms, were identified for each species.

Word frequency analysis was carried out using nVivo, version 12. For each species, sample size permitting, the ten most frequently used positive (happiness/good health) and negative (unhappiness/ill health) words were mapped to the Five Domains model of animal welfare, according to the expert opinions of two of the authors (GC and AW). The Five Domains include ‘Nutrition’, ‘Environment’, ‘Health’, ‘Behaviour’ and ‘Mental state’ (Mellor 2017). As ‘Environment’ is input-based and given the nature of the question directed to participants, this domain was not coded.

## Results and Discussion

### Participants

In total, n = 723 participants completed the survey; 238 responses were available for rabbits, 191 for guinea pigs, 163 for hamsters, 79 for rats, 22 for gerbils, 15 for mice, nine for degus and six for chinchillas.

### Barriers to, and facilitators of, providing appropriate housing

While we did not conduct statistical comparisons, Opportunity emerged as the most commonly cited barrier across all species, while Motivation and Capability were typically facilitators. The level of agreement with statements related to barriers to provision of suitable housing related to Capability is shown in Table 1.

**Table 1.** Self-reported Capability of owners (n = 723) of rabbits (*Oryctolagus cuniculus*), guinea pigs (*Cavia porcellus*), hamsters (*Cricetinae*), gerbils (*Gerbillinae*), rats (*Rattus norvegicus*), mice (*Mus musculus*), chinchillas (*Chinchilla lanigera*), and degus (*Octodon degus*)to provide appropriate housing for small mammals

Capability	Level of agreement*				
	1(stronglyagree)	2	3	4	5(stronglydisagree)
**Psychological capability**					
I did my research before acquiring my pet	75.0	18.1	2.2	3.4	1.3
I have a good understanding of the type of housing that my pet needs	85.8	13.3	0.3	0.6	0.0
I know whether my pet should be housed alone, in a pair or in a group	91.1	7.9	0.4	0.6	0.0
I know whether my pet is naturally diurnal, nocturnal or crepuscular	86.6	11.4	1.9	0.1	0.0
I know where to go for advice on pet housing	72.0	21.0	4.7	1.8	0.4
I am able to identify if the enclosure is large enough for my pet	79.8	18.3	1.5	0.4	0.0
I am aware of recommended minimum housing sizes for my pet’s species	80.3	16.1	2.1	1.5	0.0
I knew what species I was going to choose when I went to get my pet	80.1	12.1	4.0	3.1	0.6
I chose this species as I had them as a child	24.5	17.8	12.8	22.8	22.1
I choose the first enclosure I saw at the pet shop without giving it much thought	1.6	3.4	2.8	17.5	74.7
I am in the habit of cleaning my pet’s enclosure regularly	64.7	28.2	5.4	1.6	0.1
I am aware of my species’ needs and will adapt my care of them accordingly	83.2	16.1	0.7	0.0	0.0
**Physical capability**					
I am able to maintain my pet’s enclosure cleaning regime	71.9	26.1	1.3	0.6	0.1
I am physically capable of cleaning my pet’s enclosure to the standard I would like	78.7	18.4	1.7	1.1	0.1
It would be physically challenging for me to maintain a larger enclosure	3.5	9.6	15.8	38.1	33.0

* 1 = strongly agree, 2 = agree, 3 = neither agree nor disagree, 4 = disagree, 5 = strongly disagree

Most owners reported high levels of psychological and physical capability, with many stating they understood their species’ needs and housing requirements. For example, 93.1% agreed they had researched housing prior to acquiring their pet, and 99.1% reported knowing their species’ social housing needs. Owners who are well-informed about species-specific needs may be better equipped to provide suitable housing. For example, McMahon and Wigham (2020) found that owners who had higher acknowledgement of rabbit sentience were more likely to provide suitable housing and a variety of environmental enrichment types. However, it is worth noting that in the current study, we used pet owner self-reported capability, which may not reflect actual capability. As part of a larger study, we collected enclosure images and data on social housing and enrichment use. This will allow for a direct comparison between owners’ reported capability and their actual husbandry practices (Wills *et al.* in prep).

This study identified Opportunity as the most significant barrier to the provision of suitable housing for pet rabbits and rodents. The level of agreement with statements relating to Opportunity is shown in Table 2.

**Table 2.** Self-reported Opportunity of owners (n = 723) of rabbits (*Oryctolagus cuniculus*), guinea pigs (*Cavia porcellus*), hamsters (*Cricetinae*), gerbils (*Gerbillinae*), rats (*Rattus norvegicus*), mice (*Mus musculus*), chinchillas (*Chinchilla lanigera*), and degus (*Octodon degus*) to provide appropriate housing for small mammals

Opportunity	Level of agreement*				
	1(strongly agree)	2	3	4	5(strongly disagree)
**Physical opportunity**					
I can find suitable housing for my pet when I go to the pet shop	3.4	5.0	7.3	29.0	55.3
I am able to find the housing I want	17.3	35.9	12.6	20.2	13.9
I can afford the enclosure needed for my pet	36.8	40.9	13.0	6.9	2.3
I don’t have the time to work out which housing is most suitable for my pet	0.4	0.9	4.6	30.8	63.2
I don’t have the space to increase the size of my pet’s housing	6.5	28.8	12.8	29.3	22.7
**Social opportunity**					
Other people that keep this species use enclosures similar to mine	16.6	35.9	20.1	19.8	7.6
I have the support I need in helping me choose the most appropriate housing	28.9	37.9	19.2	10.9	3.1
I am able to ask for advice from professionals (e.g. vet, pet shop staff) on what housing I should be using	16.9	26.3	19.2	23.9	13.7
I am able to ask for advice from my family and friends on what type of housing I should be using	8.6	18.2	16.8	33.2	23.1
Most people whose opinion I value would approve of my pet’s current housing	61.2	31.3	4.4	1.6	1.6
My family, friends and/or society see my pet as a ‘children’s pet’	22.0	36.3	14.9	18.3	8.5
My family and friends see rabbits and small rodents as ‘starter’ pets	21.4	33.2	15.9	19.9	9.6

* 1 = strongly agree, 2 = agree, 3 = neither agree nor disagree, 4 = disagree, 5 = strongly disagree

Despite high levels of self-reported capability, many owners indicated a lack of access to appropriate housing products, particularly in pet shops. In the current study, 84.3% of respondents either disagreed or strongly disagreed that suitable housing was available at pet shops. This is consistent with earlier research indicating that enclosures for rabbits and other small mammals are frequently undersized or otherwise fail to meet welfare requirements (Harrup & Rooney 2020; Bläske *et al.* 2022; Mee *et al.* 2022). These findings suggest that the current market does not always support optimal animal welfare, likely due in part to limited evidence on species-specific housing needs and a lack of consistent guidelines. In the absence of consistent guidance or regulation, owners may be left to choose from a restricted range of housing options, some of which may not fully meet the animals’ behavioural and physical requirements. Furthermore, over one-third of participants reported insufficient space at home for provision of larger housing, reinforcing the role of environmental constraints in determining pet owner behaviour. Responses to this item were more mixed than others, possibly reflecting genuine variation in household space or the general perception of enclosure adequacy. Future analyses comparing perceived constraints with actual enclosure dimensions (collected as part of this wider study) may clarify whether those citing space limitations are already using larger enclosures or face genuine physical restrictions. Time was less frequently cited as a barrier; most owners disagreed that time limitations prevented them from selecting suitable housing. Only 1.3% of respondents agreed that they lacked the time to work out which housing was suitable, while 94% disagreed, suggesting that time was not a major limiting factor in owner decision-making. Social opportunity was also limited. Although 66.8% of participants agreed they had support in selecting housing, fewer than half felt able to ask professionals for advice, and fewer still viewed family and friends as reliable sources of advice. Notably, many respondents felt their pets were perceived by others as ‘starter pets’ or suitable only for children, attitudes which may reduce the perceived importance of providing optimal care (Rioja-Lang *et al.* 2019). Indeed, Skovlund *et al.* (2023) found that owners who viewed rabbits as starter pets were less likely to meet their basic welfare needs and housed their rabbits in more restricted enclosures.

Similar to Capability, Motivation of the surveyed pet owners was high and the level of agreement with statements relating to Motivation can be seen in Table 3.

**Table 3.** Self-reported Motivation of owners (n = 723) of rabbits (*Oryctolagus cuniculus*), guinea pigs (*Cavia porcellus*), hamsters (*Cricetinae*), gerbils (*Gerbillinae*), rats (*Rattus norvegicus*), mice (*Mus musculus*), chinchillas (*Chinchilla lanigera*), and degus (*Octodon degus*) to provide appropriate housing for small mammals

Motivation	Level of agreement*				
	1 (strongly agree)	2	3	4	5 (strongly disagree)
**Reflective motivation**					
It is my job as the pet owner to identify suitable housing for my pet	91.4	8.2	0.3	0.1	0.0
It is the job of the pet store to make sure that appropriate housing is available for sale	67.0	21.3	5.9	2.7	3.1
It is my vet’s job to inform me of what is or is not appropriate housing	15.8	27.0	27.7	22.5	7.0
I see myself as someone who cares about animal welfare	91.4	8.3	0.1	0.1	0.1
It is my responsibility to monitor my pet’s welfare and change things accordingly	93.4	6.4	0.1	0.0	0.0
I can improve my pet’s welfare by altering their enclosure	47.9	27.6	15.7	6.4	2.4
For me, providing suitable housing for my pet is easy	50.9	33.3	10.0	5.4	0.3
For me, providing housing that meets all my pet’s needs is impossible	4.2	4.3	5.9	26.7	58.9
I expect enclosures on sale in mainstream pet shops to be good enough for my pet	45.5	20.5	10.1	9.8	14.0
I expect information about my species is easily available	50.7	31.1	9.9	7.1	1.3
I expect enclosures for sale to be affordable	30.1	32.9	24.9	9.8	2.4
I have knowingly used the wrong enclosure type for my pet	3.0	7.9	3.6	28.0	57.4
If clear housing guidelines were available, I would follow them	63.7	25.8	9.9	0.6	0.0
I will buy better housing for my pet in the next year	7.3	7.6	33.0	35.9	16.3
Providing good animal welfare is a priority for me	91.1	8.6	0.1	0.1	0.0
Other aspects of husbandry (e.g. food, toys etc) are more important than housing type	4.2	4.6	49.0	35.3	6.9
The size of my pet’s cage is not important as my pet spends time outside of the cage during the day	2.8	3.8	10.9	44.1	38.4
If my pet’s enclosure is too small, they can still have good welfare	0.9	4.1	10.4	45.0	39.6
If I provide better housing, it will benefit my pet’s welfare	61.0	28.5	7.8	2.1	0.6
I am aware that if I fail to meet the needs of my pet It may be a breach of the Animal Welfare Act	65.0	26.7	4.3	3.2	0.7
**Automatic motivation**					
When I need one, I buy the same type of enclosure that I have always bought	10.1	13.9	27.7	34.7	13.6
When I buy high quality housing for my pet, I feel like I am making a difference	60.8	28.4	9.3	1.2	0.3
When I see my pet in the correct housing, it motivates me to improve their environment further	63.0	28.3	7.7	0.9	0.1
I would feel bad if I thought my pet did not have the best enclosure possible	75.7	21.6	1.4	0.8	0.4

* 1 strongly agree, 2 = agree, 3 = neither agree nor disagree, 4 = disagree, 5 = strongly disagree

Most participants expressed a strong sense of responsibility for their pet’s welfare. For example, almost all agreed that they see themselves as someone who cares about animal welfare and agreed that it is their responsibility to monitor and adjust their pet’s welfare. This suggests that interventions aiming to enhance Opportunity are likely to be well received, as the underlying Motivation already exists (Michie *et al.* 2011).

Raw response patterns by species are provided in the Supplementary material.

### Linking behavioural barriers to possible intervention strategies

Now that the sources of behaviour influencing small mammal housing behaviour in the UK have been identified, the specific barriers can be systematically linked to intervention functions shown to be effective in addressing them (Michie *et al.* 2014). For example, according to the Behaviour Change Wheel framework, barriers associated with opportunity are best addressed via the use of interventions that serve the functions of ‘Training’, ‘Restriction’, ‘Modelling’, ‘Environmental restructuring’ and ‘Enablement’ (Michie *et al.* 2011). Restricting sale of unsuitable housing (‘Restriction’) or increasing the availability of suitable housing (‘Environmental restructuring’), for instance, would make the desired behaviour more feasible and accessible to pet owners by targeting physical opportunity. Improved access to professional advice is another avenue for addressing lack of social opportunity. Given that many small pet owners are less likely to bring their animals for veterinary care (Fox & Neville 2024), expert advice at the point of sale, for example, has the potential to reach a large number of pet owners.

### Subjective animal health and welfare assessment: Word frequency analysis

This section explores owner perceptions of pet rabbit and rodent welfare using open text responses. The sample of gerbil, mouse, degu and chinchilla owners was too small to include in the analysis. Tables 4 to 7 show the top ten words that were perceived by owners to describe positive (happiness/good health) and negative (unhappiness/ill health) welfare status by species (rabbits, guinea pigs, hamsters and rats, respectively).

**Table 4.** The frequency of words used by rabbit (*Oryctolagus cuniculus*) owners (n = 231) in describing signs of happiness/good health and unhappiness/illness

Word	Mentions (n)	Examples
**Happiness/good health**		
Binkys^1^	128	*‘He runs round and binkys’*
Eating	110	*‘Eating well’*
Runs	53	*‘Hopping and running in the garden’*
Flops^2^	42	*‘Flopping onto his side’*
Plays	33	*‘Playing/interacting with us, his brother or toys’*
Zoomy^3^	25	*‘They do zoomies’*
Drinking	21	*‘Drinking well’*
Comes	20	*‘He comes running to see me’*
Relaxed	19	*‘Relaxed lying down or stretched out’*
Grooms	19	*‘Grooming other rabbit or people’*
**Unhappiness/ill health**		
Eating	138	*‘Not eating/interested in food’*
Hiding	46	*‘Runs into hiding area’*
Hunched	35	*‘Tense postures/hunched’*
Food	32	*‘Off food’*
Thumps^4^	26	*‘He thumps his feet (diva!)’*
Lethargic	24	*‘Very lethargic’*
Quiet	23	*‘Quieter than usual’*
Poops	21	*‘No poops’ ‘Poops not right – soft, runny, reduced’*
Drinking	17	*‘Not drinking’*
Sits	16	*‘Sits in one place’ ‘Sitting still’*

1 Binkys: “Spontaneous leaps into the air, sometimes with body twist (McMahon and Wigham, 2020);

2 flops = “Flopping onto their side” (McMahon & Wigham 2020);

3 zoomy: “Fast, excited running that doesn’t involve chasing to mount/bite (McMahon & Wigham 2020);

4 thumps = “thump the ground with the hind feet” (adapted from Thurston & Ottensen 2020).

**Table 5.** The frequency of words used by guinea pig (*Cavia porcellus*) owners (n = 219) in describing signs of happiness/good health and unhappiness/illness

Word	Mentions (n)	Examples
**Happiness/good health**		
Eats	107	*‘Eating habits normal’*
Popcorns^1^	71	*‘Popcorning (jumping around excitedly)’*
Active	36	*‘Physically active’*
Wheeking^2^	32	*‘Wheeking for food’*
Food	28	*‘Enthusiastic for food*
Running	27	*‘Running around’*
Drinks	24	*‘They’re drinking well*
Squeaking	19	*‘Excited squeaks’*
Eyes	16	*‘Bright eyes’*
Happy	15	*‘Happy sounds’*
**Unhappiness/ill health**		
Eating	100	*‘Not eating’*
Lethargic	39	*‘They are lethargic’*
Food	31	*‘Not interested in food*
Hiding	27	*‘Hide away’*
Weight	25	*‘If they lose weight suddenly’ ‘Losing weight’*
Drinking	23	*‘Not drinking’*
Quiet	21	*‘He goes quiet’*
Eyes	17	*‘His eyes or nose has discharge’ ‘eyes look dull’*
Hunched	16	*‘Hunched up’*
Dull	12	*‘Coat is dull’*

1 popcorns = “rapid locomotion in which the animal jumps into the air with all four limbs of the ground, often accompanied by rapid running and turning in multiple directions” (Harrup & Rooney 2020);

2 wheeking = “high-pitched vocalisation usually performed in anticipation of food or other reward” (Harrup & Rooney 2020).

**Table 6.** The frequency of words used by hamster (*Cricetinae*) owners (n = 154) in describing signs of happiness/good health and unhappiness/illness

Word	Mentions (n)	Examples
**Happiness/good health**		
Eats	75	*‘Eating and behaving normally”*
Active	64	*‘He is active and alert’*
Drinks	36	*‘He is eating and drinking’*
Wheel	35	*‘He utilises his wheel’*
Runs	31	*‘Running around’ ‘She runs in her wheel’*
Comes	27	*‘Comes out’ ‘He comes to greet me’*
Eyes	18	*‘Bright eyed’*
Foraging	16	*‘Foraging for food’ ‘Foraging, stuffing cheek pouches’*
Interacting	16	*‘She chooses to interact with me’*
Bright	15	*‘Bright and alert’*
**Unhappiness/ill health**		
Eating	100	*‘Stops eating’*
Bar	39	*‘Excessive bar chewing’*
Drinking	31	*‘Not drinking’*
Lethargic	27	*‘She’s lethargic’*
Biting	25	*‘she bite me’ ‘he bites the bar’*
Active	23	*‘She is less active’*
Chews	21	*‘Chews on plastic cage’ ‘they chew their enclosure’*
Sleeps	17	*‘Sleeping too much’*
Eyes	16	*‘Eyes dull/crusty/weeping etc’*
Food	12	*‘Food is not eaten”*

**Table 7.** The frequency of words used by rat (*Rattus norvegicus*) owners (n = 77) in describing signs of happiness/good health and unhappiness/illness

Word	Mentions (n)	Examples
**Happiness/good health**		
Boggling^1^	26	*‘Her eyes boggle’*
Eating	26	*‘He is eating normally’*
Playing	21	*‘Running around playing’*
Active	17	*‘They’re active and inquisitive’*
Drinking	16	*‘Drinking regularly’*
Interact	15	*‘Interested in interactions with me’*
Bruxing^2^	13	*‘They brux’*
Eyes	11	*‘Bright eyes’ ’Clear eyes nose etc.’*
Alert	7	*‘They’re alert’*
Bright	7	*‘Bright eyes’*
**Unhappiness/ill health**		
Eating	24	*‘Won’t eat*
Lethargic	19	*‘Lethargic and unresponsive’*
Fur	17	*‘Puffed up fur’*
Drinking	12	*‘Not eating and drinking normally’*
Eyes	11	*‘squinting eyes’ ‘eyes small’*
Food	11	*‘No interest in food’*
Squinting	11	*‘Squinting eyes’*
Breathing	10	*‘Noisy breathing’ ‘Weird breathing noises’*
Hiding	10	*‘Hides away’*
Puffed	10	*‘They have a puffed up coat’*

1 boggling = “eyes ‘popping’ in and out” (Neville *et al.* 2022);

2 bruxing = “grinding teeth without movement of the eyes” (Neville *et al.* 2022).

Word frequency analysis revealed that, overall, behavioural indicators were most commonly used to identify positive health and welfare states, while nutritional and physical signs were cited most frequently for negative states.

Interestingly, in the current study, ‘eating’ was the most commonly used word referred to when indicating unhappiness/ill health across all four examined species, and ‘eats’ was the most commonly used word referred to when indicating happiness/good health for guinea pigs and hamsters, coming second for rabbits and rats.

A reduction in eating behaviour can serve as an early indicator of underlying issues, including pain, which prey species such as rabbits and rodents are generally considered to seek to conceal, while also reducing the need for physical interaction with animals that are often difficult to handle (Carbone 2020; Venkataraman & Raajkamal 2021; James & Wills 2025). Indeed, the UK Joint Working Group on Refinement (JWGR) identified food consumption as a general indicator of welfare that can reflect the physical, physiological and psychological state of laboratory animals such as rodents (Hawkins *et al.* 2011). Furthermore, as animals like rabbits and guinea pigs eat continuously throughout the day (Gidenne *et al.* 2010; Elfers *et al.* 2021), changes to eating behaviour may be more noticeable to their owners than in species that typically eat less frequently. Together with the current study findings, this suggests that changes in eating behaviour may be a particularly salient and intuitive welfare cue for owners across multiple species and should be further explored as a potential iceberg indicator of small pet health and welfare.

In their systematic review of validated small mammal animal welfare assessment methods, Cohen and Ho (2023) identified welfare indicators shared across rabbits, guinea pigs, rats and mice, including changes in faecal output, bodyweight changes, presence of discharge from the eyes and nose, and altered food and water consumption. Several of these were identified by owners in the current study. This suggests that pet owners do possess the ability to identify relevant signs of poor health and welfare in their animals. Furthermore, this highlights the possibility that small mammal pet species may share welfare indicators that could be used to develop practical, broadly applicable monitoring tools for pet owners.

**Figure 1.**
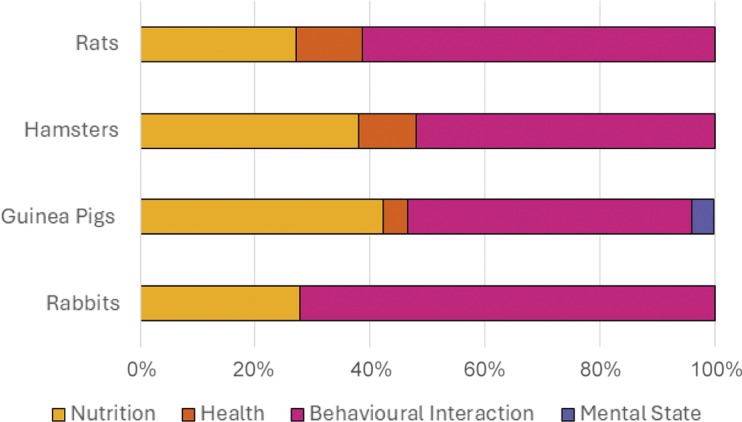
Word frequency analysis to determine signs of happiness/good health, as perceived by pet owners (n = 723), mapped to four of the five welfare domains

**Figure 2.**
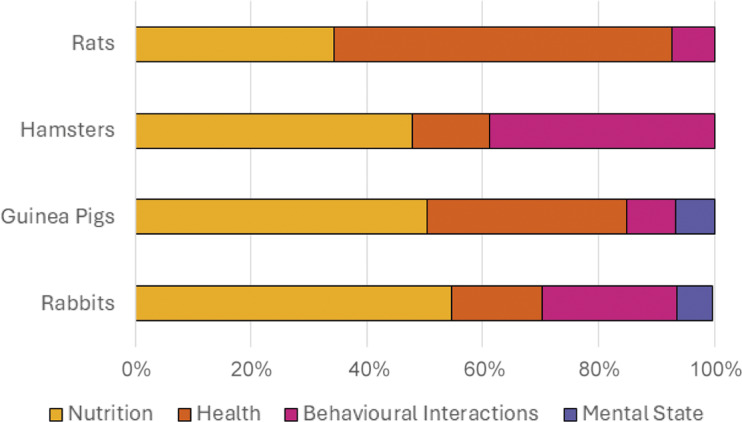
Word frequency analysis to determine signs of unhappiness/ill health, as perceived by pet owners (n = 723), mapped to four of the five welfare domains.

Figures 1 and 2 display signs of happiness/good health and unhappiness/ill health, as perceived by pet owners, mapped to four of the five welfare domains, Nutrition’, ‘Health’, ‘Behaviour’ and ‘Mental state’ (Mellor 2017). Overall, the most common domain used to signal happiness/good health across rats, hamsters, guinea pigs and rabbits was ‘Behavioural interactions’. The most common domain used to signal unhappiness/ill health in all species, apart from rats was ‘Nutrition’, with ‘Health’ being most common for rats.

### Study limitations

This study has a number of limitations. Firstly, pet owners recruited through targeted advertising may not accurately represent the typical small mammal pet owner. Instead, they are more likely to have greater species-specific knowledge and greater competence in care provision (Hedley *et al.* 2023; Fox & Neville 2024). This may limit the generalisability of the findings. Future research could utilise a point-of-purchase design, to capture information from an unbiased sample of participants. Furthermore, the use of self-reported data may not offer an accurate reflection of actual owner knowledge or practices, as participants may overestimate their capability or respond in socially desirable ways. This limitation will be addressed by comparing self-reported capability with actual husbandry practices in the same sample of participants (Wills *et al.* in prep). It is also worth noting that while multiple small mammal species were included in this study, some were underrepresented, potentially reducing the applicability of the results across all commonly kept small mammal species. In particular, further research with degu and chinchilla owners is needed. It is also possible that social desirability played a role in this study. In particular, participants may have felt compelled to respond favourably to very direct and value-laden items such as “Providing good animal welfare is a priority for me”. One solution would be to use indirect questions (Ried *et al.* 2022), for instance, “Providing good animal welfare is a priority for small mammal pet owners”. This has been used previously in animal welfare research, with findings indicating differences between directly and indirectly worded questions (e.g. Lusk & Norwood 2010). Follow-up work will compare self-reported responses to submitted images of housing and enrichment, allowing us to assess the extent of social desirability bias. Finally, this survey collected information from UK pet owners which may not translate directly to other countries, or to other contexts, such as laboratory settings.

### Animal welfare implications

This study identified Opportunity as the principal barrier to providing suitable housing for small mammal pets, with most owners reporting challenges accessing appropriate enclosures. Capability and Motivation were reported as high, suggesting that many owners are both willing and able to provide good welfare, but are constrained by external factors. The findings also show that owners use observable cues, particularly eating behaviour, to assess animal health and welfare. These cues may offer a practical means of early detection of health or welfare concerns, especially in prey species, such as rabbits and rodents, which are known to conceal signs of pain or illness. Addressing the gap between owner motivation and the availability of suitable housing will require targeted interventions designed with industry stakeholders. Improvements in the design, promotion and accessibility of welfare-compliant housing, in conjunction with guidance that reflects species-specific needs, could support better husbandry across a wide range of small mammal pets.

## Conclusion

This study used the COM-B model to examine the behavioural factors influencing the provision of suitable housing for small mammal pets. Lack of Opportunity was identified as the primary barrier for UK small mammal pet owners, while Capability and Motivation acted as facilitators. The results of the current study can now be mapped to suitable intervention strategies to increase the use of species-appropriate housing solutions. Indicators of ill health and poor welfare reported by pet owners often related to nutrition, particularly changes in eating behaviour. Further research should explore the potential of eating behaviour as an iceberg indicator of health and welfare issues in small mammal pets.

## Supporting information

Carroll et al. supplementary material Carroll et al. supplementary material

## Supplementary material

The supplementary material for this article can be found at http://doi.org/10.1017/awf.2025.10034.

## References

[r1] Allaby M 2003 A Dictionary of Zoology. Oxford University Press: Oxford, UK.

[r2] Baradad VP and Mugabushaka AM 2015 *Corpus specific stop words to improve the textual analysis in scientometrics.* ISSI Conference, June 2015. https://www.issi-society.org/proceedings/issi_2015/0999.pdf (accessed 3 August 2025).

[r3] Bläske A, Schwarzer A, Ebner MV, Gerbig H, Reese S, Erhard M and Wöhr AC 2022 Evaluation of small mammal pet supplies offered in German retail under animal welfare aspects. PLOS One 17: e0262658. 10.1371/journal.pone.0262658 35108305 PMC8809526

[r4] Blue Cross 2024 *Blue Cross Pet Census 2024.* https://www.bluecross.org.uk/pet-census (accessed 30 July 2025).

[r5] Cane J, O’Connor D and Michie S 2012 Validation of the theoretical domains framework for use in behaviour change and implementation research. Implementation Science 7: 37. 10.1186/1748-5908-7-3 22530986 PMC3483008

[r6] Carbone L 2020 Do “prey species” hide their pain? Implications for ethical care and use of laboratory animals. Journal of Applied Animal Ethics Research 2: 216–236. 10.1163/25889567-BJA10001

[r7] Carroll GA and Groarke JM 2019 The importance of the social sciences in reducing tail biting prevalence in pigs. Animals 9: 591. 10.3390/ani9090591 31438625 PMC6770512

[r8] Carroll GA, Groarke JM and Graham-Wisener L 2021 Human behaviour change models for improving animal welfare. Bridging Research Disciplines to Advance Animal Welfare Science: A Practical Guide pp 91–106. CABI: Wallingford, UK.

[r9] Cohen S and Ho C 2023 Review of rat (*Rattus norvegicus*), mouse (*Mus musculus*), guinea pig (*Cavia porcellus*) and rabbit (*Oryctolagus cuniculus*) indicators for welfare assessment. Animals 13(13): 2167. 10.3390/ani13132167 37443965 PMC10339950

[r10] Cornish A, Jamieson J, Raubenheimer D and McGreevy P 2019 Applying the behavioural change wheel to encourage higher welfare food choices. Animals 9: 524. 10.3390/ani9080524 31382457 PMC6719989

[r11] Cowdell F and Dyson J 2019 How is the theoretical domains framework applied to developing health behaviour interventions? A systematic search and narrative synthesis. BMC Public Health 19: 10. 10.1186/s12889-019-7442-5 31455327 PMC6712870

[r12] Díaz-Berciano C and Gallego-Agundez M 2024 Abandonment and rehoming of rabbits and rodents in Madrid (Spain): a retrospective study (2008–2021). Journal of Applied Animal Welfare Science 27: 712–722. 10.1080/10888705.2022.2162342 36585785

[r13] Dixon LM, Hardiman JR and Cooper JJ 2010 The effects of spatial restriction on the behavior of rabbits (*Oryctolagus cuniculus*). Journal of Veterinary Behavior 5: 302–308. 10.1016/j.jveb.2010.07.002

[r14] EFSA Panel on Animal Health and Welfare 2012 Statement on the use of animal‐based measures to assess the welfare of animals. EFSA Journal 10: 2767. 10.2903/j.efsa.2012.2767

[r15] Elfers K, Armbrecht Y and Mazzuoli-Weber G 2021 Good to know: Baseline data on feed intake, fecal pellet output and intestinal transit time in guinea pig as a frequently used model in gastrointestinal research. Animals 11(6): 1593. 10.3390/ani11061593 34071498 PMC8227794

[r16] Espinosa García-San Román J, Quesada-Canales Ó, Arbelo Hernández M, Déniz Suárez S and Castro-Alonso A 2023 Veterinary education and wtraining on non-traditional companion animals, exotic, zoo, and wild animals: Concepts review and challenging perspective on zoological medicine. Veterinary Sciences 10(5): 357. 10.3390/vetsci10050357 37235440 PMC10222605

[r17] Feng X and Behar-Horenstein LS 2019 Maximizing NVivo utilities to analyze open-ended responses. The Qualitative Report 24: 563–571. 10.46743/2160-3715/2019.3692

[r18] Fox A and Neville V 2024 Burrowing for answers: Investigating Syrian hamster welfare through owner surveys. Veterinary Record 195(9): e4534. 10.1002/vetr.4534 39113341

[r19] Fulton EA, Brown KE, Kwah KL and Wild S 2016 StopApp: using the behaviour change wheel to develop an app to increase uptake and attendance at NHS Stop Smoking Services. Healthcare 4(2): 31. 10.3390/healthcare4020031 27417619 PMC4934584

[r20] Grant RA, Montrose VT and Wills AP 2017 ExNOTic: Should we be keeping exotic pets? Animals 7: 47. 10.3390/ani7060047 28629177 PMC5483610

[r21] Gidenne T, Lebas F and Fortun-Lamothe L 2010 Chapter 13. Feeding behaviour of rabbits. In: De Blas C and Wiseman J (eds) Nutrition of the Rabbit, Second Edition pp 233–252. CAB International: Wallingford, UK.

[r22] Gilhofer EM, Hebesberger DV, Waiblinger S, Künzel F, Rouha-Mülleder C, Mariti C and Windschnurer I 2024 Husbandry conditions and welfare state of pet chinchillas (*Chinchilla lanigera*) and caretakers’ perceptions of stress and emotional closeness to their animals. Animals 14: 3155. 10.3390/ani14213155 39518878 PMC11544953

[r23] Harrup AJ and Rooney NJ 2020 Current welfare state of pet guinea pigs in the UK. Veterinary Record 186: 282. 10.1136/vr.105632 32054719

[r24] Hawkins P, Morton DB, Burman O, Dennison N, Honess P and Jennings M 2011 A guide to defining and implementing protocols for the welfare assessment of laboratory animals: eleventh report of the BVAAWF/FRAME/RSPCA/UFAW Joint Working Group on Refinement. Laboratory Animals 45: 1–13. 10.1258/la.2010.010031 21123303

[r25] Hedley JE, Pettitt A and Abeyesinghe SM 2023 Preliminary investigation into the housing of dwarf hamsters. Veterinary Record 193(4): e3170. 10.1002/vetr.3170 37527402

[r26] James L and Wills AP 2025 Perception and utilisation of veterinary services by rodent owners in the United Kingdom. Veterinary Record 196(8): e4958. 10.1002/vetr.4958 39865427 PMC12007489

[r27] Linderholm A and Larson G 2013 The role of humans in facilitating and sustaining coat colour variation in domestic animals. Seminars in Cell & Developmental Biology 24: 587–593. 10.1016/j.semcdb.2013.03.015 23567209

[r28] Lonstein JS and De Vries GJ 2000 Sex differences in the parental behavior of rodents. Neuroscience & Biobehavioral Reviews 24: 669–686. 10.1016/S0149-7634(00)00036-1 10940441

[r29] Lusk JL and Norwood FB 2010 Direct versus indirect questioning: an application to the well-being of farm animals. Social Indicators Research 96(3): 551–565. 10.1007/s11205-009-9492-z

[r30] Mazhary H and Hawkins P 2019 Applying the 3Rs: A case study on evidence and perceptions relating to rat cage height in the UK. Animals 9: 1104. 10.3390/ani9121104 31835402 PMC6940895

[r31] McLaughlin A and Strunk A 2016 Common emergencies in small rodents, hedgehogs, and sugar gliders. Veterinary Clinics of North America: Exotic Animal Practice 19: 465–499. 10.1016/j.cvex.2016.01.008 27131160

[r32] McMahon SA and Wigham E 2020 ‘All Ears’: A questionnaire of 1516 owner perceptions of the mental abilities of pet rabbits, subsequent resource provision, and the effect on welfare. Animals 10: 1730. 10.3390/ani10101730 32977692 PMC7598668

[r33] Mee G, Tipton E, Oxley JA and Westgarth C 2022 Owner demographic factors are associated with suitable pet rabbit housing provision in the United Kingdom. Veterinary Record 190: e1736. 10.1002/vetr.1736 35661365

[r34] Mellor DJ 2017 Operational details of the five domains model and its key applications to the assessment and management of animal welfare. Animals 7(8): 60. 10.3390/ani7080060 28792485 PMC5575572

[r35] Michie S, Atkins L and West R 2014 The Behaviour Change Wheel. A Guide to Designing Interventions. Silverback Publishing: Sutton, UK.

[r36] Michie S, Van Stralen MM and West R 2011 The behaviour change wheel: A new method for characterising and designing behaviour change interventions. Implementation Science 6: 42. 10.1186/1748-5908-6-42 21513547 PMC3096582

[r37] Mitchell MA 2009 History of exotic pets. Manual of Exotic Pet Practice pp 1–3. WB Saunders: St Louis, MO, USA.

[r38] Neesam S 2015 *Welfare guidance for proper care of cavies.* http://www.britishcavycouncil.org.uk/Welfare/ (accessed 1 August 2025).

[r39] Neville V, Mounty J, Benato L, Hunter K, Mendl M and Paul ES 2022 Thinking outside the lab: Can studies of pet rats inform pet and laboratory rat welfare? Applied Animal Behaviour Science 246: 105507. 10.1016/j.applanim.2021.105507

[r40] O’Neill DG, Kim K, Brodbelt DC, Church DB, Pegram C and Baldrey V 2022 Demography, disorders and mortality of pet hamsters under primary veterinary care in the United Kingdom in 2016. Journal of Small Animal Practice 63: 747–755. 10.1111/jsap.13527 35732354 PMC9796486

[r41] Onwuegbuzie AJ and Leech NL 2007 Validity and qualitative research: An oxymoron? Quality & Quantity 41: 233–249. 10.1007/s11135-006-9000-3

[r43] Ried L, Eckerd S and Kaufmann L 2022 Social desirability bias in PSM surveys and behavioral experiments: Considerations for design development and data collection. Journal of Purchasing and Supply Management 28(1): 100743. 10.1016/j.pursup.2021.100743

[r44] Rioja‐Lang F, Bacon H, Connor M and Dwyer CM 2019 Rabbit welfare: Determining priority welfare issues for pet rabbits using a modified Delphi method. Veterinary Record Open 6: e000363. 10.1136/vetreco-2019-000363 31903189 PMC6924855

[r45] Robinson NJ, Dean RS, Cobb M and Brennan ML 2014 Consultation length in first opinion small animal practice. Veterinary Record 175: 486. 10.1136/vr.102713 PMC4251166 25261270

[r46] Rooney NJ, Blackwell EJ, Mullan SM, Saunders R, Baker PE and Hill JM 2014 The current state of welfare, housing and husbandry of the English pet rabbit population. BMC Research Notes 7: 1–13. 10.1186/1756-0500-7-942 25532711 PMC4307134

[r47] Ross AP, Norvelle A, Choi DC, Walton JC, Albers HE and Huhman KL 2017 Social housing and social isolation: Impact on stress indices and energy balance in male and female Syrian hamsters (*Mesocricetus auratus*). Physiology & Behavior 177: 264–269. 10.1016/j.physbeh.2017.05.015 28511867 PMC5538356

[r48] Saré RM, Lemons A and Smith CB 2021 Behavior testing in rodents: Highlighting potential confounds affecting variability and reproducibility. Brain Sciences 11: 522. 10.3390/brainsci11040522 33924037 PMC8073298

[r49] Schneidewind S, Lesch R, Heizmann V and Windschnurer I 2024 Exploring pet rat care: A comprehensive survey of husbandry, health, behavior, and the associations between caretaker attitudes, attachment, and husbandry practices. Journal of Veterinary Behavior 76: 49–61. 10.1016/j.jveb.2024.06.009

[r50] Skovlund CR, Forkman B, Lund TB, Mistry BG, Nielsen SS and Sandøe P 2023 Perceptions of the rabbit as a low investment ’starter pet’ lead to negative impacts on its welfare: Results of two Danish surveys. Animal Welfare 32: e45. 10.1017/awf.2023.41 38487438 PMC10936283

[r51] Thurston S and Ottesen JL 2020 The rabbit. Animal-centric Care and Management pp 135–148. CRC Press: USA.

[r52] Truelove S, Vanderloo LM, Tucker P, Di Sebastiano KM and Faulkner G 2020 The use of the behaviour change wheel in the development of ParticipACTION’s physical activity app. Preventive Medicine Reports 20: 101224. 10.1016/j.pmedr.2020.101224 33134041 PMC7585152

[r53] UK Government 2006 *Animal Welfare Act 2006.* https://www.legislation.gov.uk/ukpga/2006/45/contents (accessed 20 May 2025).

[r54] UK Pet Food 2024 *2024 Annual Report.* https://ukpetfood-reports.co.uk/ (accessed 30 July 2025).

[r55] Venkataraman K and Raajkamal BS 2021 Clinical examination of laboratory rodents and rabbits. Essentials of Laboratory Animal Science: Principles and Practices pp 521–539. Elsevier: London, UK.

[r56] Whittal A, Atkins L and Herber OR 2021 What the guide does not tell you: reflections on and lessons learned from applying the COM-B behavior model for designing real life interventions. Translational Behavioral Medicine 11(5): 1122–1126. 10.1093/tbm/ibaa116 33200792

[r57] Wills A and Holt S 2020 Confidence of veterinary surgeons in the United Kingdom in treating and diagnosing exotic pet species. Veterinary Record 186: e20. 10.1136/vr.105664 PMC7365564 32015163

[r58] Wills AP 2020 Impact of husbandry on the welfare of pet guinea pigs in the UK. Veterinary Record 186(9): 279–281. 10.1136/vr.m743 32139628

[r59] Yeates J and Baumans V 2019 Rodents. In: McMillan FD (ed) Companion Animal Care and Welfare: The UFAW Companion Animal Handbook pp 145–156. CABI: Boston, MA, USA.

