# Supplementary material for: Small mammal owners’ experiences of housing challenges and animal welfare: A COM-B and word frequency analysis
Source: Anim Welf. 2025 Aug 21;34:e59. doi: 10.1017/awf.2025.10034 (PMC12451400; doi:10.1017/awf.2025.10034)
Supplement: Carroll et al. supplementary material [file S0962728625100341sup001.pdf]

Self-reported Capability of owners (N = 723) of rabbits (n=238), guinea pigs (n=191), hamsters (n=163), gerbils (n =22), rats (n = 79), mice (n = 15), chinchillas (n = 6), and degus (n = 9) to provide appropriate housing for small mammals. Result are reported for each Capability, Opportunity and Motivation for each species examined, separately.

Note the small sample of responses from mouse, chinchilla and degu owners.

**Capability- Chinchillas**	**Level of agreement***				
	**1**	**2**	**3**	**4**	**5**
**Psychological capability**					
I did my research before acquiring my pet	83.3	16.7	0.0	0.0	0.0
I have a good understanding of the type of housing that my pet needs	100.0	0.0	0.0	0.0	0.0
I know whether my pet should be housed alone, in a pair or in a group	100.0	0.0	0.0	0.0	0.0
I know whether my pet is naturally diurnal, nocturnal or crepuscular	100.0	0.0	0.0	0.0	0.0
I know where to go for advice on pet housing	83.3	16.7	0.0	0.0	0.0
I am able to identify if the enclosure is large enough for my pet	66.7	33.3	0.0	0.0	0.0
I am aware of recommended minimum housing sizes for my pet's species	66.7	33.3	0.0	0.0	0.0
I knew what species I was going to choose when I went to get my pet	83.3	16.7	0.0	0.0	0.0
I chose this species as I had them as a child *	33.3	0.0	0.0	0.0	66.7
I choose the first enclosure I saw at the pet shop without giving it much thought	0.0	0.0	0.0	0.0	100.0
I am in the habit of cleaning my pet's enclosure regularly	83.3	16.7	0.0	0.0	0.0
I am aware of my species needs and will adapt my care of them accordingly	100.0	0.0	0.0	0.0	0.0
**Physical capability**					
I am able to maintain my pet's enclosure cleaning regime	66.7	33.3	0.0	0.0	0.0
I am physically capable of cleaning my pet's enclosure to the standard I would like	83.3	16.7	0.0	0.0	0.0
It would be physically challenging for me to maintain a larger enclosure	0.0	0.0	0.0	20.0	80.0

Self-reported Capability of owners (N = 723) of rabbits (n=238), guinea pigs (n=191), hamsters (n=163), gerbils (n =22), rats (n = 79), mice (n = 15), chinchillas (n = 6), and degus (n = 9) to provide appropriate housing for small mammals. Result are reported for each Capability, Opportunity and Motivation for each species examined, separately.

Note the small sample of responses from mouse, chinchilla and degu owners.

Capability- Degus	Level of agreement*				
	1	2	3	4	5
**Psychological capability**					
I did my research before acquiring my pet	55.6	11.1	11.1	11.1	11.1
I have a good understanding of the type of housing that my pet needs	88.9	11.1	0.0	0.0	0.0
I know whether my pet should be housed alone, in a pair or in a group	88.9	11.1	0.0	0.0	0.0
I know whether my pet is naturally diurnal, nocturnal or crepuscular	10.00.0	0.0	0.0	0.0	0.0
I know where to go for advice on pet housing	66.7	33.3	0.0	0.0	0.0
I am able to identify if the enclosure is large enough for my pet	66.7	22.2	0.0	11.1	0.0
I am aware of recommended minimum housing sizes for my pet’s species	55.6	0.0	33.3	11.1	0.0
I knew what species I was going to choose when I went to get my pet	62.5	12.5	12.5	12.5	0.0
I chose this species as I had them as a child	0.0	0.0	12.5	25	62.5
I choose the first enclosure I saw at the pet shop without giving it much thought	14.3	0.0	0.0	28.6	57.1
I am in the habit of cleaning my pet’s enclosure regularly	44.4	44.4	11.1	0.0	0.0
I am aware of my species needs and will adapt my care of them accordingly	66.7	33.3	0.0	0.0	0.0
**Physical capability**					
I am able to maintain my pet’s enclosure cleaning regime	77.8	22.2	0.0	0.0	0.0
I am physically capable of cleaning my pet’s enclosure to the standard I would like	66.7	33.3	0.0	0.0	0.0
It would be physically challenging for me to maintain a larger enclosure	0.0	25	0.0	62.5	12.5

Self-reported Capability of owners (N = 723) of rabbits (n=238), guinea pigs (n=191), hamsters (n=163), gerbils (n =22), rats (n = 79), mice (n = 15), chinchillas (n = 6), and degus (n = 9) to provide appropriate housing for small mammals. Result are reported for each Capability, Opportunity and Motivation for each species examined, separately.

Note the small sample of responses from mouse, chinchilla and degu owners.

Capability- Gerbils	Level of agreement*				
	1	2	3	4	5
**Psychological capability**					
I did my research before acquiring my pet	76.2	14.3	9.5	0.0	0.0
I have a good understanding of the type of housing that my pet needs	95.5	4.5	0.0	0.0	0.0
I know whether my pet should be housed alone, in a pair or in a group	95.5	4.5	0.0	0.0	0.0
I know whether my pet is naturally diurnal, nocturnal or crepuscular	90.0.9	9.1	0.0	0.0	0.0
I know where to go for advice on pet housing	81.8	18.2	0.0	0.0	0.0
I am able to identify if the enclosure is large enough for my pet	86.4	13.6	0.0	0.0	0.0
I am aware of recommended minimum housing sizes for my pet's species	86.4	13.6	0.0	0.0	0.0
I knew what species I was going to choose when I went to get my pet	75.0	25.0	0.0	0.0	0.0
I chose this species as I had them as a child	21.1	15.8	10.5	21.1	31.6
I choose the first enclosure I saw at the pet shop without giving it much thought	0.0	11.1	0.0	27.8	61.1
I am in the habit of cleaning my pet's enclosure regularly	55	30.0	10.0	5.0.0	0.0
I am aware of my species needs and will adapt my care of them accordingly	81	19	0.0	0.0	0.0
**Physical capability**					
I am able to maintain my pet's enclosure cleaning regime	81	14.3	4.8	0.0	0.0
I am physically capable of cleaning my pet's enclosure to the standard I would like	90.9	9.1	0.0	0.0	0.0
It would be physically challenging for me to maintain a larger enclosure	9.1	0.0	22.7	22.7	45.5

Self-reported Capability of owners (N = 723) of rabbits (n=238), guinea pigs (n=191), hamsters (n=163), gerbils (n =22), rats (n = 79), mice (n = 15), chinchillas (n = 6), and degus (n = 9) to provide appropriate housing for small mammals. Result are reported for each Capability, Opportunity and Motivation for each species examined, separately.

Note the small sample of responses from mouse, chinchilla and degu owners.

**Capability- Guinea Pigs**	**Level of agreement***				
	**1**	**2**	**3**	**4**	**5**
**Psychological capability**					
I did my research before acquiring my pet	76.5	17.1	1.6	4.3	0.5
I have a good understanding of the type of housing that my pet needs	82.5	15.8	1.1	0.5	0.0
I know whether my pet should be housed alone, in a pair or in a group	94.2	5.2	0.0	0.5	0.0
I know whether my pet is naturally diurnal, nocturnal or crepuscular	80.0	16.3	3.2	0.5	0.0
I know where to go for advice on pet housing	70.9	21.7	5.3	2.1	0.0
I am able to identify if the enclosure is large enough for my pet	78.0	18.8	2.6	0.5	0.0
I am aware of recommended minimum housing sizes for my pet's species	77.0	18.3	3.7	1.0	0.0
I knew what species I was going to choose when I went to get my pet	84.0	11.2	2.1	2.7	0.0
I chose this species as I had them as a child	34.1	15.9	10.4	18.9	20.7
I choose the first enclosure I saw at the pet shop without giving it much thought	0.6	1.8	1.8	18.2	77.6
I am in the habit of cleaning my pet's enclosure regularly	76.4	21.5	1.6	0.5	0.0
I am aware of my species needs and will adapt my care of them accordingly	81.2	17.8	1.0	0.0	0.0
**Physical capability**					
I am able to maintain my pet's enclosure cleaning regime	72.3	26.2	0.5	0.5	0.5
I am physically capable of cleaning my pet's enclosure to the standard I would like	76.8	20	1.6	1.6	0.0
It would be physically challenging for me to maintain a larger enclosure	0.5	15.2	21.2	41.3	21.7

Self-reported Capability of owners (N = 723) of rabbits (n=238), guinea pigs (n=191), hamsters (n=163), gerbils (n =22), rats (n = 79), mice (n = 15), chinchillas (n = 6), and degus (n = 9) to provide appropriate housing for small mammals. Result are reported for each Capability, Opportunity and Motivation for each species examined, separately.

Note the small sample of responses from mouse, chinchilla and degu owners.

**Capability- Hamsters**	**Level of agreement***				
	**1**	**2**	**3**	**4**	**5**
**Psychological capability**					
I did my research before acquiring my pet	79.1	14.1	1.2	4.9	0.6
I have a good understanding of the type of housing that my pet needs	86.5	12.9	0.0	0.6	0.0
I know whether my pet should be housed alone, in a pair or in a group	96.9	2.5	0.0	0.6	0.0
I know whether my pet is naturally diurnal, nocturnal or crepuscular	94.5	5.5	0.0	0.0	0.0
I know where to go for advice on pet housing	71.8	22.7	2.5	2.5	0.6
I am able to identify if the enclosure is large enough for my pet	79.8	18.4	1.8	0.0	0.0
I am aware of recommended minimum housing sizes for my pet’s species	81.6	16.6	0.6	1.2	0.0
I knew what species I was going to choose when I went to get my pet	80.3	10.8	3.2	5.1	0.6
I chose this species as I had them as a child	27.4	21.5	10.4	17.8	23.0
I choose the first enclosure I saw at the pet shop without giving it much thought	3.3	4.0	2.6	15.9	74.2
I am in the habit of cleaning my pet’s enclosure regularly	43.8	38.3	13.6	3.7	0.6
I am aware of my species needs and will adapt my care of them accordingly	80.4	18.4	1.2	0.0	0.0
**Physical capability**					
I am able to maintain my pet’s enclosure cleaning regime	72.4	25.8	1.8	0.0	0.0
I am physically capable of cleaning my pet’s enclosure to the standard I would like	82.2	14.7	1.2	1.8	0.0
It would be physically challenging for me to maintain a larger enclosure	4.3	6.8	7.5	39.1	42.2

Self-reported Capability of owners (N = 723) of rabbits (n=238), guinea pigs (n=191), hamsters (n=163), gerbils (n =22), rats (n = 79), mice (n = 15), chinchillas (n = 6), and degus (n = 9) to provide appropriate housing for small mammals. Result are reported for each Capability, Opportunity and Motivation for each species examined, separately.

Note the small sample of responses from mouse, chinchilla and degu owners.

Capability- Mice	Level of agreement*				
	1	2	3	4	5
**Psychological capability**					
I did my research before acquiring my pet	93.3	0.0	6.7	0.0	0.0
I have a good understanding of the type of housing that my pet needs	93.3	6.7	0.0	0.0	0.0
I know whether my pet should be housed alone, in a pair or in a group	93.3	6.7	0.0	0.0	0.0
I know whether my pet is naturally diurnal, nocturnal or crepuscular	93.3	6.7	0.0	0.0	0.0
I know where to go for advice on pet housing	80.0	20.0	0.0	0.0	0.0
I am able to identify if the enclosure is large enough for my pet	86.7	13.3	0.0	0.0	0.0
I am aware of recommended minimum housing sizes for my pet's species	80.0	13.3	6.7	0.0	0.0
I knew what species I was going to choose when I went to get my pet	86.7	6.7	6.7	0.0	0.0
I chose this species as I had them as a child	27.3	9.1	9.1	27.3	27.3
I choose the first enclosure I saw at the pet shop without giving it much thought	0.0	0.0	14.3	14.3	71.4
I am in the habit of cleaning my pet's enclosure regularly	33.3	60.0	0.0	6.7	0.0
I am aware of my species needs and will adapt my care of them accordingly	93.3	6.7	0.0	0.0	0.0
**Physical capability**					
I am able to maintain my pet's enclosure cleaning regime	53.3	40.0	6.7	0.0	0.0
I am physically capable of cleaning my pet's enclosure to the standard I would like	80.0	20.0	0.0	0.0	0.0
It would be physically challenging for me to maintain a larger enclosure	0.0	7.1	14.3	57.1	21.4

Self-reported Capability of owners (N = 723) of rabbits (n=238), guinea pigs (n=191), hamsters (n=163), gerbils (n =22), rats (n = 79), mice (n = 15), chinchillas (n = 6), and degus (n = 9) to provide appropriate housing for small mammals. Result are reported for each Capability, Opportunity and Motivation for each species examined, separately.

Note the small sample of responses from mouse, chinchilla and degu owners.

**Capability- Rabbits**	**Level of agreement***				
	**1**	**2**	**3**	**4**	**5**
**Psychological capability**					
I did my research before acquiring my pet	68.5	24.1	2.6	2.6	2.2
I have a good understanding of the type of housing that my pet needs	85.2	13.9	0.0	0.0.8	0.0
I know whether my pet should be housed alone, in a pair or in a group	82.1	15.7	1.3	0.0.9	0.0
I know whether my pet is naturally diurnal, nocturnal or crepuscular	84.9	11.8	3.4	0.0	0.0
I know where to go for advice on pet housing	68.4	21.3	7.1	2.2	0.0.9
I am able to identify if the enclosure is large enough for my pet	78.4	19.8	1.3	0.0.4	0.0
I am aware of recommended minimum housing sizes for my pet's species	81.7	13.2	2.6	2.6	0.0
I knew what species I was going to choose when I went to get my pet	75.8	13.7	6.2	3.1	1.3
I chose this species as I had them as a child	19.5	19	18	25.5	18
I choose the first enclosure I saw at the pet shop without giving it much thought	1.7	5.0	3.9	17.8	71.7
I am in the habit of cleaning my pet's enclosure regularly	75.7	21.2	2.7	0.4	0.0
I am aware of my species needs and will adapt my care of them accordingly	85.2	14.3	0.4	0.0	0.0
**Physical capability**					
I am able to maintain my pet's enclosure cleaning regime	73.7	25	0.9	0.4	0.0
I am physically capable of cleaning my pet's enclosure to the standard I would like	79.5	17.0	2.2	0.9	0.4
It would be physically challenging for me to maintain a larger enclosure	3.6	7.7	16.4	35.4	36.9

Self-reported Capability of owners (N = 723) of rabbits (n=238), guinea pigs (n=191), hamsters (n=163), gerbils (n =22), rats (n = 79), mice (n = 15), chinchillas (n = 6), and degus (n = 9) to provide appropriate housing for small mammals. Result are reported for each Capability, Opportunity and Motivation for each species examined, separately.

Note the small sample of responses from mouse, chinchilla and degu owners.

**Capability- Rats**	**Level of agreement***				
	**1**	**2**	**3**	**4**	**5**
**Psychological capability**					
I did my research before acquiring my pet	79.7	16.5	1.3	1.3	1.3
I have a good understanding of the type of housing that my pet needs	88.6	11.4	0.0	0.0	0.0
I know whether my pet should be housed alone, in a pair or in a group	96.2	3.8	0.0	0.0	0.0
I know whether my pet is naturally diurnal, nocturnal or crepuscular	86.1	13.9	0.0	0.0	0.0
I know where to go for advice on pet housing	81.0	15.2	3.8	0.0	0.0
I am able to identify if the enclosure is large enough for my pet	87.3	12.7	0.0	0.0	0.0
I am aware of recommended minimum housing sizes for my pet’s species	83.5	16.5	0.0	0.0	0.0
I knew what species I was going to choose when I went to get my pet	84.8	10.1	3.8	1.3	0.0
I chose this species as I had them as a child	11.5	16.4	9.8	36.1	26.2
I choose the first enclosure I saw at the pet shop without giving it much thought	0.0	1.4	1.4	16.7	80.6
I am in the habit of cleaning my pet’s enclosure regularly	57	36.7	5.1	1.3	0.0
I am aware of my species needs and will adapt my care of them accordingly	87.3	12.7	0.0	0.0	0.0
**Physical capability**					
I am able to maintain my pet’s enclosure cleaning regime	65.8	30.4	1.3	2.5	0.0
I am physically capable of cleaning my pet’s enclosure to the standard I would like	70.9	26.6	2.5	0.0	0.0
It would be physically challenging for me to maintain a larger enclosure	7.8	9.1	19.5	35.1	28.6

Self-reported Capability of owners (N = 723) of rabbits (n=238), guinea pigs (n=191), hamsters (n=163), gerbils (n =22), rats (n = 79), mice (n = 15), chinchillas (n = 6), and degus (n = 9) to provide appropriate housing for small mammals. Result are reported for each Capability, Opportunity and Motivation for each species examined, separately.

Note the small sample of responses from mouse, chinchilla and degu owners.

Opportunity- Chinchillas	Level of agreement*				
	1	2	3	4	5
**Physical opportunity**					
I can find suitable housing for my pet when I go to the pet shop	0.0	0.0	16.7	0.0	83.3
I am able to find the housing I want	40.0	20.0	40.0	0.0	0.0
I can afford the enclosure needed for my pet	66.7	16.7	0.0	16.7	0.0
I don't have the time to work out which housing is most suitable for my pet	0.0	0.0	0.0	50.0	50.0
I don't have the space to increase the size of my pet's housing	0.0	16.7	16.7	0.0	66.7
**Social opportunity**					
Other people that keep this species use enclosures similar to mine	16.7	66.7	16.7	0.0	0.0
I have the support I need in helping me choose the most appropriate housing	16.7	16.7	50.0	0.0	16.7
I am able to ask for advice from professionals (e.g. vet, pet shop staff) on what housing I should be using	33.3	16.7	0.0	16.7	33.3
I am able to ask for advice from my family and friends on what type of housing I should be using	33.3	33.3	16.7	0.0	16.7
Most people whose opinion I value would approve of my pet's current housing	66.7	16.7	0.0	0.0	16.7
My family, friends and/or society see my pet as a 'children's pet'	0.0	16.7	16.7	50.0	16.7
My family and friends see rabbits and small rodents as 'starter' pets	0.0	16.7	16.7	16.7	50.0

Opportunity-Degus	Level of agreement*				
	1	2	3	4	5
**Physical opportunity**					
I can find suitable housing for my pet when I go to the pet shop	0.0	0.0	11.1	33.3	55.6
I am able to find the housing I want	22.2	11.1	11.1	33.3	22.2
I can afford the enclosure needed for my pet	66.7	11.1	11.1	11.1	0.0
I don’t have the time to work out which housing is most suitable for my pet	0.0	0.0	11.1	33.3	55.6
I don’t have the space to increase the size of my pet’s housing	0.0	44.4	11.1	22.2	22.2
**Social opportunity**					
Other people that keep this species use enclosures similar to mine	11.1	66.7	0.0	11.1	11.1
I have the support I need in helping me choose the most appropriate housing	44.4	22.2	0.0	33.3	0.0
I am able to ask for advice from professionals (e.g. vet, pet shop staff) on what housing I should be using	44.4	11.1	0.0	33.3	11.1
I am able to ask for advice from my family and friends on what type of housing I should be using	22.2	11.1	11.1	33.3	22.2
Most people whose opinion I value would approve of my pet’s current housing	55.6	22.2	0.0	22.2	0.0
My family, friends and/or society see my pet as a ‘children’s pet’	11.1	0.0	11.1	33.3	44.4
My family and friends see rabbits and small rodents as ‘starter’ pets	25.0	12.5	12.5	25.0	25.0

Self-reported Capability of owners (N = 723) of rabbits (n=238), guinea pigs (n=191), hamsters (n=163), gerbils (n =22), rats (n = 79), mice (n = 15), chinchillas (n = 6), and degus (n = 9) to provide appropriate housing for small mammals. Result are reported for each Capability, Opportunity and Motivation for each species examined, separately.

Note the small sample of responses from mouse, chinchilla and degu owners.

**Opportunity- Gerbils**	**Level of agreement***				
	**1**	**2**	**3**	**4**	**5**
**Physical opportunity**					
I can find suitable housing for my pet when I go to the pet shop	9.1	18.2	9.1	9.1	54.5
I am able to find the housing I want	36.4	31.8	9.1	13.6	9.1
I can afford the enclosure needed for my pet	45.5	45.5	9.1	0.0	0.0
I don't have the time to work out which housing is most suitable for my pet	0.0	5.0	0.0	25.0	70.0
I don't have the space to increase the size of my pet's housing	5.0	20.0	10.0	45.0	20.0
**Social opportunity**					
Other people that keep this species use enclosures similar to mine	23.8	42.9	14.3	9.5	9.5
I have the support I need in helping me choose the most appropriate housing	33.3	52.4	4.8	9.5	0.0
I am able to ask for advice from professionals (e.g. vet, pet shop staff) on what housing I should be using	27.8	11.1	16.7	16.7	27.8
I am able to ask for advice from my family and friends on what type of housing I should be using	10.0	5.0	30.0	25	30.0
Most people whose opinion I value would approve of my pet's current housing	52.6	36.8	10.5	0.0	0.0
My family, friends and/or society see my pet as a 'children's pet'	42.1	26.3	15.8	15.8	0.0
My family and friends see rabbits and small rodents as 'starter' pets	26.3	31.6	26.3	15.8	0.0

Self-reported Capability of owners (N = 723) of rabbits (n=238), guinea pigs (n=191), hamsters (n=163), gerbils (n =22), rats (n = 79), mice (n = 15), chinchillas (n = 6), and degus (n = 9) to provide appropriate housing for small mammals. Result are reported for each Capability, Opportunity and Motivation for each species examined, separately.

Note the small sample of responses from mouse, chinchilla and degu owners.

Opportunity- Guinea Pigs	Level of agreement*				
	**1**	**2**	**3**	**4**	**5**
**Physical opportunity**					
I can find suitable housing for my pet when I go to the pet shop	4.3	6.5	5.4	34.2	49.5
I am able to find the housing I want	20.0.2	48.4	9.6	13.3	8.5
I can afford the enclosure needed for my pet	40.0	45.7	8	4.3	1.6
I don't have the time to work out which housing is most suitable for my pet	0.0	0.0.5	4.9	37.7	56.8
I don't have the space to increase the size of my pet's housing	6.9	42.3	14.3	25.1	11.4
**Social opportunity**					
Other people that keep this species use enclosures similar to mine	18.7	45.5	18.2	13.4	4.3
I have the support I need in helping me choose the most appropriate housing	25.3	39.6	25.8	6	3.3
I am able to ask for advice from professionals (e.g. vet, pet shop staff) on what housing I should be using	16.7	33.3	22.8	21.1	6.1
I am able to ask for advice from my family and friends on what type of housing I should be using	8.7	18	18.6	37.2	17.4
Most people whose opinion I value would approve of my pet's current housing	56.4	38.8	3.7	1.1	0.0
My family, friends and/or society see my pet as a 'children's pet'	22.3	39.4	14.4	18.6	5.3
My family and friends see rabbits and small rodents as 'starter' pets	21.4	38	18.2	16.6	5.9

Self-reported Capability of owners (N = 723) of rabbits (n=238), guinea pigs (n=191), hamsters (n=163), gerbils (n =22), rats (n = 79), mice (n = 15), chinchillas (n = 6), and degus (n = 9) to provide appropriate housing for small mammals. Result are reported for each Capability, Opportunity and Motivation for each species examined, separately.

Note the small sample of responses from mouse, chinchilla and degu owners.

Opportunity- Hamsters	Level of agreement*				
	**1**	**2**	**3**	**4**	**5**
**Physical opportunity**					
I can find suitable housing for my pet when I go to the pet shop	1.9	4.3	6.8	25.5	61.5
I am able to find the housing I want	10.7	32.1	13.2	32.7	11.3
I can afford the enclosure needed for my pet	31.7	42.9	14.3	9.3	1.9
I don’t have the time to work out which housing is most suitable for my pet	0.6	0.6	7.5	25.6	65.6
I don’t have the space to increase the size of my pet’s housing	7.7	26.3	12.2	28.8	25
**Social opportunity**					
Other people that keep this species use enclosures similar to mine	14.6	34.4	18.5	26.8	5.7
I have the support I need in helping me choose the most appropriate housing	30.4	38	11.4	15.8	4.4
I am able to ask for advice from professionals (e.g. vet, pet shop staff) on what housing I should be using	11.9	17	15.7	30.8	24.5
I am able to ask for advice from my family and friends on what type of housing I should be using	5.2	20.3	11.8	33.3	29.4
Most people whose opinion I value would approve of my pet’s current housing	29.8	42.2	12.4	11.8	3.7
My family, friends and/or society see my pet as a ‘children’s pet’	28.9	35.8	11.3	17	6.9
My family and friends see rabbits and small rodents as ‘starter’ pets	14.6	34.4	18.5	26.8	5.7

Self-reported Capability of owners (N = 723) of rabbits (n=238), guinea pigs (n=191), hamsters (n=163), gerbils (n =22), rats (n = 79), mice (n = 15), chinchillas (n = 6), and degus (n = 9) to provide appropriate housing for small mammals. Result are reported for each Capability, Opportunity and Motivation for each species examined, separately.

Note the small sample of responses from mouse, chinchilla and degu owners.

Opportunity- Mice	Level of agreement*				
	**1**	**2**	**3**	**4**	**5**
**Physical opportunity**					
I can find suitable housing for my pet when I go to the pet shop	13.3	0.0	6.7	20.0	60.0
I am able to find the housing I want	13.3	53.3	0.0	13.3	20.0
I can afford the enclosure needed for my pet	6.7	66.7	20.0	0.0	6.7
I don't have the time to work out which housing is most suitable for my pet	6.7	0.0	0.0	26.7	66.7
I don't have the space to increase the size of my pet's housing	6.7	13.3	6.7	40.0	33.3
**Social opportunity**					
Other people that keep this species use enclosures similar to mine	15.4	38.5	30.0.8	15.4	0.0
I have the support I need in helping me choose the most appropriate housing	20.0	60.0	13.3	6.7	0.0
I am able to ask for advice from professionals (e.g. vet, pet shop staff) on what housing I should be using	13.3	26.7	13.3	40.0	6.7
I am able to ask for advice from my family and friends on what type of housing I should be using	13.3	20.0	6.7	33.3	26.7
Most people whose opinion I value would approve of my pet's current housing	66.7	20.0	13.3	0.0	0.0
My family, friends and/or society see my pet as a 'children's pet'	35.7	35.7	14.3	7.1	7.1
My family and friends see rabbits and small rodents as 'starter' pets	33.3	26.7	6.7	13.3	20.0

Self-reported Capability of owners (N = 723) of rabbits (n=238), guinea pigs (n=191), hamsters (n=163), gerbils (n =22), rats (n = 79), mice (n = 15), chinchillas (n = 6), and degus (n = 9) to provide appropriate housing for small mammals. Result are reported for each Capability, Opportunity and Motivation for each species examined, separately.

Note the small sample of responses from mouse, chinchilla and degu owners.

**Opportunity- Rabbits**	**Level of agreement***				
	**1**	**2**	**3**	**4**	**5**
**Physical opportunity**					
I can find suitable housing for my pet when I go to the pet shop	2.6	4	6.6	30.0	56.8
I am able to find the housing I want	12.7	22.4	17.1	22.9	24.9
I can afford the enclosure needed for my pet	35.6	34.7	15.3	10.0.4	4.0
I don't have the time to work out which housing is most suitable for my pet	0.5	1.5	3.5	30.7	63.9
I don't have the space to increase the size of my pet's housing	7.2	21.7	12.2	30.0	28.9
**Social opportunity**					
Other people that keep this species use enclosures similar to mine	8.4	20.6	26.6	29.4	15
I have the support I need in helping me choose the most appropriate housing	26.6	33	23.2	13.8	3.4
I am able to ask for advice from professionals (e.g. vet, pet shop staff) on what housing I should be using	18.8	30.3	22	19.3	9.6
I am able to ask for advice from my family and friends on what type of housing I should be using	9.7	17	18.9	32	22.3
Most people whose opinion I value would approve of my pet's current housing	64.9	27.6	4.4	1.3	1.8
My family, friends and/or society see my pet as a 'children's pet'	20.2	39.5	13.7	15.9	10.7
My family and friends see rabbits and small rodents as 'starter' pets	20.5	32.1	14.1	22.2	11.1

Self-reported Capability of owners (N = 723) of rabbits (n=238), guinea pigs (n=191), hamsters (n=163), gerbils (n =22), rats (n = 79), mice (n = 15), chinchillas (n = 6), and degus (n = 9) to provide appropriate housing for small mammals. Result are reported for each Capability, Opportunity and Motivation for each species examined, separately.

Note the small sample of responses from mouse, chinchilla and degu owners.

**Opportunity- Rats**	**Level of agreement***				
	**1**	**2**	**3**	**4**	**5**
**Physical opportunity**					
I can find suitable housing for my pet when I go to the pet shop	3.8	3.8	12.7	30.4	49.4
I am able to find the housing I want	29.1	50.6	8.9	7.6	3.8
I can afford the enclosure needed for my pet	39.2	40.5	17.7	1.3	1.3
I don’t have the time to work out which housing is most suitable for my pet	0.0	0.0	2.6	26.3	71.1
I don’t have the space to increase the size of my pet’s housing	2.7	24.3	13.5	35.1	24.3
**Social opportunity**					
Other people that keep this species use enclosures similar to mine	36.7	49.4	12.7	1.3	0.0
I have the support I need in helping me choose the most appropriate housing	40.3	41.6	14.3	3.9	0.0
I am able to ask for advice from professionals (e.g. vet, pet shop staff) on what housing I should be using	15.8	23.7	15.8	27.6	17.1
I am able to ask for advice from my family and friends on what type of housing I should be using	6.9	20.8	16.7	31.9	23.6
Most people whose opinion I value would approve of my pet’s current housing	65.4	33.3	0.0	0.0	1.3
My family, friends and/or society see my pet as a ‘children’s pet’	5.3	14.7	25.3	37.3	17.3
My family and friends see rabbits and small rodents as ‘starter’ pets	6.5	24.7	24.7	28.6	15.6

Self-reported Capability of owners (N = 723) of rabbits (n=238), guinea pigs (n=191), hamsters (n=163), gerbils (n =22), rats (n = 79), mice (n = 15), chinchillas (n = 6), and degus (n = 9) to provide appropriate housing for small mammals. Result are reported for each Capability, Opportunity and Motivation for each species examined, separately.

Note the small sample of responses from mouse, chinchilla and degu owners.

Motivation- Chinchillas	Level of agreement*				
	**1**	**2**	**3**	**4**	**5**
**Reflective motivation**					
It is my job as the pet owner to identify suitable housing for my pet	100.0	0.0	0.0	0.0	0.0
It is the job of the pet store to make sure that appropriate housing is available for sale	20.0	0.0	20.0	40.0	20.0
It is my vet's job to inform me of what is or is not appropriate housing	16.7	0.0	33.3	33.3	16.7
I see myself as someone who cares about animal welfare	100.0	0.0	0.0	0.0	0.0
It is my responsibility to monitor my pets' welfare and change things accordingly	100.0	0.0	0.0	0.0	0.0
I can improve my pet's welfare by altering their enclosure	50.0	16.7	16.7	16.7	0.0
For me, providing suitable housing for my pet is easy	66.7	16.7	0.0	16.7	0.0
For me, providing housing that meets all my pet's needs is impossible	16.7	0.0	16.7	16.7	50.0
I expect enclosures on sale in mainstream pet shops to be good enough for my pet	16.7	16.7	33.3	33.3	0.0
I expect information about my species is easily available	33.3	16.7	33.3	0.0	16.7
I expect enclosures for sale to be affordable	0.0	50.0	16.7	33.3	0.0
I have knowingly used the wrong enclosure type for my pet	0.0	0.0	33.3	16.7	50.0
If clear housing guidelines were available, I would follow them	50	50.0	0.0	0.0	0.0
I will buy better housing for my pet in the next year	0.0	0.0	50.0	33.3	16.7
Providing good animal welfare is a priority for me	100.0	0.0	0.0	0.0	0.0
Other aspects of husbandry (e.g. food, toys etc.) are more important than housing type	0.0	16.7	66.7	16.7	0.0
The size of my pet's cage is not important as my pet spends time outside of the cage during the day	0.0	0.0	0.0	25.0	75.0
If my pet's enclosure is too small, they can still have good welfare	0.0	0.0	16.7	16.7	66.7
If I provide better housing, it will benefit my pet's welfare	60.0	40.0	0.0	0.0	0.0
I am aware that if I fail to meet the needs of my pet It may be a breach of the Animal Welfare Act	83.3	16.7	0.0	0.0	0.0
**Automatic motivation**					
When I need one, I buy the same type of enclosure that I have always bought	0.0	1	2	3	4
When I buy high quality housing for my pet, I feel like I am making a difference	33.3	0.0	33.3	33.3	0.0
When I see my pet in the correct housing, it motivates me to improve their environment further	50.0	33.3	16.7	0.0	0.0

Self-reported Capability of owners (N = 723) of rabbits (n=238), guinea pigs (n=191), hamsters (n=163), gerbils (n =22), rats (n = 79), mice (n = 15), chinchillas (n = 6), and degus (n = 9) to provide appropriate housing for small mammals. Result are reported for each Capability, Opportunity and Motivation for each species examined, separately.

Note the small sample of responses from mouse, chinchilla and degu owners.

I would feel bad if I thought my pet did not have the best enclosure possible	50.0	16.7	33.3	0.0	0.0

Self-reported Capability of owners (N = 723) of rabbits (n=238), guinea pigs (n=191), hamsters (n=163), gerbils (n =22), rats (n = 79), mice (n = 15), chinchillas (n = 6), and degus (n = 9) to provide appropriate housing for small mammals. Result are reported for each Capability, Opportunity and Motivation for each species examined, separately.

Note the small sample of responses from mouse, chinchilla and degu owners.

Motivation- Degus	Level of agreement*				
	**1**	**2**	**3**	**4**	**5**
**Reflective motivation**					
It is my job as the pet owner to identify suitable housing for my pet	88.9	11.1	0.0	0.0	0.0
It is the job of the pet store to make sure that appropriate housing is available for sale	88.9	11.1	0.0	0.0	0.0
It is my vet's job to inform me of what is or is not appropriate housing	25.0	12.5	12.5	37.5	12.5
I see myself as someone who cares about animal welfare	88.9	11.1	0.0	0.0	0.0
It is my responsibility to monitor my pets' welfare and change things accordingly	88.9	11.1	0.0	0.0	0.0
I can improve my pet's welfare by altering their enclosure	66.7	11.1	22.2	0.0	0.0
For me, providing suitable housing for my pet is easy	77.8	0.0	22.2	0.0	0.0
For me, providing housing that meets all my pet's needs is impossible	22.2	0.0	11.2	11.1	55.6
I expect enclosures on sale in mainstream pet shops to be good enough for my pet	55.6	11.1	22.2	11.1	0.0
I expect information about my species is easily available	66.7	11.1	11.1	11.1	0.0
I expect enclosures for sale to be affordable	44.4	11.1	33.3	11.1	0.0
I have knowingly used the wrong enclosure type for my pet	0.0	0.0	11.1	44.4	44.4
If clear housing guidelines were available, I would follow them	77.8	11.1	11.1	0.0	0.0
I will buy better housing for my pet in the next year	25	0.0	37.5	25	12.5
Providing good animal welfare is a priority for me	88.9	11.1	0.0	0.0	0.0
Other aspects of husbandry (e.g. food, toys etc.) are more important than housing type	11.1	0.0	44.4	44.4	0.0
The size of my pet's cage is not important as my pet spends time outside of the cage during the day	0.0	0.0	11.1	55.6	33.3
If my pet's enclosure is too small, they can still have good welfare	0.0	0.0	0.0	55.6	44.4
If I provide better housing, it will benefit my pet's welfare	66.7	22.2	0.0	11.1	0.0
I am aware that if I fail to meet the needs of my pet It may be a breach of the Animal Welfare Act	88.9	11.1	0.0	0.0	0.0
**Automatic motivation**					
When I need one, I buy the same type of enclosure that I have always bought	28.6	0.0	14.3	28.6	28.6
When I buy high quality housing for my pet, I feel like I am making a difference	62.5	37.5	0.0	0.0	0.0

Self-reported Capability of owners (N = 723) of rabbits (n=238), guinea pigs (n=191), hamsters (n=163), gerbils (n =22), rats (n = 79), mice (n = 15), chinchillas (n = 6), and degus (n = 9) to provide appropriate housing for small mammals. Result are reported for each Capability, Opportunity and Motivation for each species examined, separately.

Note the small sample of responses from mouse, chinchilla and degu owners.

When I see my pet in the correct housing, it motivates me to improve their environment further	77.8	11.1	11.1	0.0	0.0
I would feel bad if I thought my pet did not have the best enclosure possible	88.9	11.1	0.0	0.0	0.0

Motivation- Gerbils	Level of agreement*				
	1	2	3	4	5
**Reflective motivation**					
It is my job as the pet owner to identify suitable housing for my pet	90.9	9.1	0.0	0.0	0.0
It is the job of the pet store to make sure that appropriate housing is available for sale	77.3	18.2	4.5	0.0	0.0
It is my vet's job to inform me of what is or is not appropriate housing	25	25	30.0	20.0	0.0
I see myself as someone who cares about animal welfare	95.2	4.8	0.0	0.0	0.0
It is my responsibility to monitor my pets' welfare and change things accordingly	95.2	4.8	0.0	0.0	0.0
I can improve my pet's welfare by altering their enclosure	52.4	23.8	19	0.0	4.8
For me, providing suitable housing for my pet is easy	47.6	42.9	4.8	4.8	0.0
For me, providing housing that meets all my pet's needs is impossible	4.8	0.0	0.0	38.1	57.1
I expect enclosures on sale in mainstream pet shops to be good enough for my pet	63.6	18.2	0.0	4.5	13.6
I expect information about my species is easily available	54.5	27.3	4.5	9.1	4.5
I expect enclosures for sale to be affordable	40.9	31.8	18.2	4.5	4.5
I have knowingly used the wrong enclosure type for my pet	4.5	9.1	4.5	27.3	54.5
If clear housing guidelines were available, I would follow them	81.8	18.2	0.0	0.0	0.0
I will buy better housing for my pet in the next year	12.5	0.0	25	37.5	25
Providing good animal welfare is a priority for me	95.5	4.5	0.0	0.0	0.0
Other aspects of husbandry (e.g. food, toys etc.) are more important than housing type	4.5	0.0	50.0	31.8	13.6
The size of my pet's cage is not important as my pet spends time outside of the cage during the day	5.3	5.3	10.5	31.6	47.4
If my pet's enclosure is too small, they can still have good welfare	4.5	4.5	18.2	36.4	36.4

Self-reported Capability of owners (N = 723) of rabbits (n=238), guinea pigs (n=191), hamsters (n=163), gerbils (n =22), rats (n = 79), mice (n = 15), chinchillas (n = 6), and degus (n = 9) to provide appropriate housing for small mammals. Result are reported for each Capability, Opportunity and Motivation for each species examined, separately.

Note the small sample of responses from mouse, chinchilla and degu owners.

If I provide better housing, it will benefit my pet's welfare	57.9	26.3	10.5	5.3	0.0
I am aware that if I fail to meet the needs of my pet It may be a breach of the Animal Welfare Act	50.0	31.8	9.1	9.1	0.0
**Automatic motivation**					
When I need one, I buy the same type of enclosure that I have always bought	10.5	0.0	21.1	52.6	15.8
When I buy high quality housing for my pet, I feel like I am making a difference	66.7	19	14.3	0.0	0.0
When I see my pet in the correct housing, it motivates me to improve their environment further	61.9	28.6	9.5	0.0	0.0
I would feel bad if I thought my pet did not have the best enclosure possible	66.7	33.3	0.0	0.0	0.0

Motivation- Guinea pigs	Level of agreement*				
	**1**	**2**	**3**	**4**	**5**
**Reflective motivation**					
It is my job as the pet owner to identify suitable housing for my pet	89.5	10.0	0.0	0.0.5	0.0
It is the job of the pet store to make sure that appropriate housing is available for sale	63	23.8	7.9	1.6	3.7
It is my vet's job to inform me of what is or is not appropriate housing	10.6	29.1	28.6	25.4	6.3
I see myself as someone who cares about animal welfare	90.5	8.9	0.0	0.0.5	0.0
It is my responsibility to monitor my pets' welfare and change things accordingly	92	7.5	0.5	0.0	0.0
I can improve my pet's welfare by altering their enclosure	43	29	16.7	8.6	2.7
For me, providing suitable housing for my pet is easy	49.5	39.9	8	2.7	0.0
For me, providing housing that meets all my pet's needs is impossible	3.3	4.4	3.9	26.5	61.9
I expect enclosures on sale in mainstream pet shops to be good enough for my pet	44	23	10.5	11.5	11.0
I expect information about my species is easily available	46.1	43.5	6.8	3.1	0.5
I expect enclosures for sale to be affordable	30.0	38.4	24.7	5.8	1.1
I have knowingly used the wrong enclosure type for my pet	2.1	7.5	4.3	28.3	57.8
If clear housing guidelines were available, I would follow them	59.4	29.4	11.1	0.0	0.0
I will buy better housing for my pet in the next year	6.5	7.3	30.9	37.4	17.9

Self-reported Capability of owners (N = 723) of rabbits (n=238), guinea pigs (n=191), hamsters (n=163), gerbils (n =22), rats (n = 79), mice (n = 15), chinchillas (n = 6), and degus (n = 9) to provide appropriate housing for small mammals. Result are reported for each Capability, Opportunity and Motivation for each species examined, separately.

Note the small sample of responses from mouse, chinchilla and degu owners.

Providing good animal welfare is a priority for me	90.0	9.5	0.0	0.5	0.0
Other aspects of husbandry (e.g. food, toys etc.) are more important than housing type	3.2	4.7	45.8	39.5	6.8
The size of my pet's cage is not important as my pet spends time outside of the cage during the day	1.2	3.0	12.1	52.1	31.5
If my pet's enclosure is too small, they can still have good welfare	1.1	6.0	13.6	49.5	29.9
If I provide better housing, it will benefit my pet's welfare	50.6	33.9	10.7	3.0	1.8
I am aware that if I fail to meet the needs of my pet It may be a breach of the Animal Welfare Act	60.4	30.5	5.9	3.2	0.0
**Automatic motivation**					
When I need one, I buy the same type of enclosure that I have always bought	13	22.4	26.1	29.2	9.3
When I buy high quality housing for my pet, I feel like I am making a difference	49.2	37.0	12.7	1.1	0.0
When I see my pet in the correct housing, it motivates me to improve their environment further	51.4	37.8	9.7	1.1	0.0
I would feel bad if I thought my pet did not have the best enclosure possible	72.8	23.0	2.6	1.0	0.5

Motivation- Hamsters	Level of agreement*				
	**1**	**2**	**3**	**4**	**5**
**Reflective motivation**					
It is my job as the pet owner to identify suitable housing for my pet	92	7.4	0.0.6	0.0	0.0
It is the job of the pet store to make sure that appropriate housing is available for sale	75.8	19.3	1.9	1.9	1.2
It is my vet's job to inform me of what is or is not appropriate housing	20.4	26.5	27.2	20.4	5.6
I see myself as someone who cares about animal welfare	90.8	9.2	0.0	0.0	0.0
It is my responsibility to monitor my pets' welfare and change things accordingly	93.2	6.8	0.0	0.0	0.0
I can improve my pet's welfare by altering their enclosure	56.6	30.8	8.2	2.5	1.9
For me, providing suitable housing for my pet is easy	48.5	31.9	9.8	9.2	0.6
For me, providing housing that meets all my pet's needs is impossible	1.9	5.6	9.3	31.5	51.9
I expect enclosures on sale in mainstream pet shops to be good enough for my pet	53.1	14.4	7.5	8.8	16.3
I expect information about my species is easily available	62.6	20.9	7.4	8	1.2

Self-reported Capability of owners (N = 723) of rabbits (n=238), guinea pigs (n=191), hamsters (n=163), gerbils (n =22), rats (n = 79), mice (n = 15), chinchillas (n = 6), and degus (n = 9) to provide appropriate housing for small mammals. Result are reported for each Capability, Opportunity and Motivation for each species examined, separately.

Note the small sample of responses from mouse, chinchilla and degu owners.

I expect enclosures for sale to be affordable	36.4	29.6	16	14.8	3.1
I have knowingly used the wrong enclosure type for my pet	4.4	11.3	4.4	29.6	50.3
If clear housing guidelines were available, I would follow them	69.2	21.4	7.5	1.9	0.0
I will buy better housing for my pet in the next year	8.1	8.9	37.1	33.1	12.9
Providing good animal welfare is a priority for me	91.4	8.6	0.0	0.0	0.0
Other aspects of husbandry (e.g. food, toys etc.) are more important than housing type	2.5	4.3	50.0	35.8	7.4
The size of my pet's cage is not important as my pet spends time outside of the cage during the day	0.0	1.3	5.1	36.1	57.6
If my pet's enclosure is too small, they can still have good welfare	0.0.6	6.2	9.3	41.4	42.6
If I provide better housing, it will benefit my pet's welfare	69.7	23.9	5.2	1.3	0.0
I am aware that if I fail to meet the needs of my pet It may be a breach of the Animal Welfare Act	66.7	24.7	4.3	2.5	1.9
**Automatic motivation**					
When I need one, I buy the same type of enclosure that I have always bought	6.2	12.3	20.8	40.0	20.8
When I buy high quality housing for my pet, I feel like I am making a difference	72	21.7	5.6	0.6	0.0
When I see my pet in the correct housing, it motivates me to improve their environment further	75.6	19.4	5	0.0	0.0
I would feel bad if I thought my pet did not have the best enclosure possible	78.3	19.9	0.0	0.0.6	1.2

Motivation- Mice	Level of agreement*				
	1	2	3	4	5
**Reflective motivation**					
It is my job as the pet owner to identify suitable housing for my pet	100.0	0.0	0.0	0.0	0.0
It is the job of the pet store to make sure that appropriate housing is available for sale	66.7	13.3	13.3	0.0	6.7
It is my vet's job to inform me of what is or is not appropriate housing	14.3	28.6	42.9	7.1	7.1
I see myself as someone who cares about animal welfare	93.3	6.7	0.0	0.0	0.0
It is my responsibility to monitor my pets' welfare and change things accordingly	100.0	0.0	0.0	0.0	0.0
I can improve my pet's welfare by altering their enclosure	35.7	35.7	14.3	14.3	0.0

Self-reported Capability of owners (N = 723) of rabbits (n=238), guinea pigs (n=191), hamsters (n=163), gerbils (n =22), rats (n = 79), mice (n = 15), chinchillas (n = 6), and degus (n = 9) to provide appropriate housing for small mammals. Result are reported for each Capability, Opportunity and Motivation for each species examined, separately.

Note the small sample of responses from mouse, chinchilla and degu owners.

For me, providing suitable housing for my pet is easy	26.7	60.0	6.7	6.7	0.0
For me, providing housing that meets all my pet's needs is impossible	6.7	6.7	6.7	33.3	46.7
I expect enclosures on sale in mainstream pet shops to be good enough for my pet	26.7	46.7	6.7	0.0	20.0
I expect information about my species is easily available	20.0	33.3	26.7	13.3	6.7
I expect enclosures for sale to be affordable	26.7	33.3	26.7	6.7	6.7
I have knowingly used the wrong enclosure type for my pet	6.7	6.7	0.0	40.0	46.7
If clear housing guidelines were available, I would follow them	46.7	33.3	13.3	6.7	0.0
I will buy better housing for my pet in the next year	9.1	0.0	45.5	45.5	0.0
Providing good animal welfare is a priority for me	93.3	6.7	0.0	0.0	0.0
Other aspects of husbandry (e.g. food, toys etc.) are more important than housing type	13.3	0.0	46.7	33.3	6.7
The size of my pet's cage is not important as my pet spends time outside of the cage during the day	6.7	0.0	0.0	33.3	60.0
If my pet's enclosure is too small, they can still have good welfare	7.1	0.0	7.1	50.0	35.7
If I provide better housing, it will benefit my pet's welfare	64.3	28.6	0.0	0.0	7.1
I am aware that if I fail to meet the needs of my pet It may be a breach of the Animal Welfare Act	60.0	33.3	0.0	0.0	6.7
**Automatic motivation**					
When I need one, I buy the same type of enclosure that I have always bought	14.3	0.0	21.4	57.1	7.1
When I buy high quality housing for my pet, I feel like I am making a difference	73.3	26.7	0.0	0.0	0.0
When I see my pet in the correct housing, it motivates me to improve their environment further	86.7	13.3	0.0	0.0	0.0
I would feel bad if I thought my pet did not have the best enclosure possible	60.0	33.3	6.7	0.0	0.0

Motivation- Rabbits	Level of agreement*				
	1	2	3	4	5
**Reflective motivation**					
It is my job as the pet owner to identify suitable housing for my pet	90.2	9.4	0.4	0.0	0.0
It is the job of the pet store to make sure that appropriate housing is available for sale	63.9	22.7	6.4	3.9	3.0

Self-reported Capability of owners (N = 723) of rabbits (n=238), guinea pigs (n=191), hamsters (n=163), gerbils (n =22), rats (n = 79), mice (n = 15), chinchillas (n = 6), and degus (n = 9) to provide appropriate housing for small mammals. Result are reported for each Capability, Opportunity and Motivation for each species examined, separately.

Note the small sample of responses from mouse, chinchilla and degu owners.

It is my vet's job to inform me of what is or is not appropriate housing	18.6	28.3	25.3	20.7	7.2
I see myself as someone who cares about animal welfare	90.3	9.3	0.4	0.0	0.0
It is my responsibility to monitor my pets' welfare and change things accordingly	93.3	6.7	0.0	0.0	0.0
I can improve my pet's welfare by altering their enclosure	43.6	25.0	19.1	9.3	2.9
For me, providing suitable housing for my pet is easy	53.8	27.5	11.4	6.8	0.4
For me, providing housing that meets all my pet's needs is impossible	6.2	4.4	4.0	24.0	61.3
I expect enclosures on sale in mainstream pet shops to be good enough for my pet	43.5	20.0	10.9	9.6	16.1
I expect information about my species is easily available	51.1	28.5	12.3	7.2	0.9
I expect enclosures for sale to be affordable	27.1	30.0.1	31.0	8.3	3.5
I have knowingly used the wrong enclosure type for my pet	3.7	6.9	2.8	24.4	62.2
If clear housing guidelines were available, I would follow them	63.8	23.3	12.9	0.0	0.0
I will buy better housing for my pet in the next year	4.3	9.6	35.7	34.8	15.7
Providing good animal welfare is a priority for me	90.7	8.9	0.0.4	0.0	0.0
Other aspects of husbandry (e.g. food, toys etc.) are more important than housing type	6.0	4.3	47.0	35.3	7.3
The size of my pet's cage is not important as my pet spends time outside of the cage during the day	7.6	8.1	14.6	41.1	28.6
If my pet's enclosure is too small, they can still have good welfare	0.4	2.7	8.0	41.1	47.8
If I provide better housing, it will benefit my pet's welfare	62.2	27	9.7	1.1	0.0
I am aware that if I fail to meet the needs of my pet It may be a breach of the Animal Welfare Act	68.5	24.7	2.6	3.8	0.4
**Automatic motivation**					
When I need one, I buy the same type of enclosure that I have always bought	5.7	11.3	34	31.9	17
When I buy high quality housing for my pet, I feel like I am making a difference	57.1	29.1	11.1	1.6	1.1
When I see my pet in the correct housing, it motivates me to improve their environment further	59.5	28.4	9.8	1.9	0.5
I would feel bad if I thought my pet did not have the best enclosure possible	76.6	21.3	0.9	1.3	0.0

Self-reported Capability of owners (N = 723) of rabbits (n=238), guinea pigs (n=191), hamsters (n=163), gerbils (n =22), rats (n = 79), mice (n = 15), chinchillas (n = 6), and degus (n = 9) to provide appropriate housing for small mammals. Result are reported for each Capability, Opportunity and Motivation for each species examined, separately.

Note the small sample of responses from mouse, chinchilla and degu owners.

Motivation - Rats	Level of agreement*				
	1	2	3	4	5
**Reflective motivation**					
It is my job as the pet owner to identify suitable housing for my pet	96.2	3.8	0.0	0.0	0.0
It is the job of the pet store to make sure that appropriate housing is available for sale	65.4	20.5	6.4	2.6	5.1
It is my vet's job to inform me of what is or is not appropriate housing	7.6	22.8	31.6	26.6	11.4
I see myself as someone who cares about animal welfare	96.2	3.8	0.0	0.0	0.0
It is my responsibility to monitor my pets' welfare and change things accordingly	96.2	3.8	0.0	0.0	0.0
I can improve my pet's welfare by altering their enclosure	52	26.7	18.7	1.3	1.3
For me, providing suitable housing for my pet is easy	51.9	35.4	12.7	0.0	0.0
For me, providing housing that meets all my pet's needs is impossible	1.3	2.6	9.1	23.4	63.6
I expect enclosures on sale in mainstream pet shops to be good enough for my pet	39.2	25.3	12.7	10.1	12.7
I expect information about my species is easily available	40.5	34.2	11.4	12.7	1.3
I expect enclosures for sale to be affordable	24.1	35.4	26.6	13.9	0.0
I have knowingly used the wrong enclosure type for my pet	0.0	5.1	1.3	30.8	62.8
If clear housing guidelines were available, I would follow them	59.6	33.8	6.8	0.0	0.0
I will buy better housing for my pet in the next year	10.9	6.5	17.4	41.3	23.9
Providing good animal welfare is a priority for me	92.3	7.7	0.0	0.0	0.0
Other aspects of husbandry (e.g. food, toys etc.) are more important than housing type	2.6	7.7	60.3	25.6	3.8
The size of my pet's cage is not important as my pet spends time outside of the cage during the day	0.0	1.3	14.1	55.1	29.5
If my pet's enclosure is too small, they can still have good welfare	0.0	1.3	11.4	55.7	31.6
If I provide better housing, it will benefit my pet's welfare	63	30.1	4.1	2.7	0.0
I am aware that if I fail to meet the needs of my pet It may be a breach of the Animal Welfare Act	63.3	27.8	6.3	2.5	0.0
**Automatic motivation**					
When I need one, I buy the same type of enclosure that I have always bought	14.9	11.9	35.8	34.3	3
When I buy high quality housing for my pet, I feel like I am making a difference	70.0.1	22.1	5.2	2.6	0.0

Self-reported Capability of owners (N = 723) of rabbits (n=238), guinea pigs (n=191), hamsters (n=163), gerbils (n =22), rats (n = 79), mice (n = 15), chinchillas (n = 6), and degus (n = 9) to provide appropriate housing for small mammals. Result are reported for each Capability, Opportunity and Motivation for each species examined, separately.

Note the small sample of responses from mouse, chinchilla and degu owners.

When I see my pet in the correct housing, it motivates me to improve their environment further	69.6	29.1	1.3	0.0	0.0
I would feel bad if I thought my pet did not have the best enclosure possible	78.5	19	2.5	0.0	0.0
